# Remolding laccase for whole-cell and in vivo modulation of dopamine signal

**DOI:** 10.1126/sciadv.ady3842

**Published:** 2025-10-22

**Authors:** Xiaoti Yang, Shilong Fan, Jing Liu, Shuli Chen, Wenjie Wu, Xiling Chen, Wenliang Ji, Shuxin Li, Yifei Xue, Xinjie Sun, Ming Wang, Ji Liu, Fei Wu, Ping Yu, Lanqun Mao

**Affiliations:** ^1^ College of Chemistry, Beijing Normal University, Beijing 100875, China.; ^2^ Beijing National Laboratory for Molecular Sciences, Key Laboratory of Analytical Chemistry for Living Biosystems, Institute of Chemistry, Chinese Academy of Sciences (CAS), Beijing 100190, China.; ^3^ Beijing Advanced Innovation Center for Structural Biology, School of Life Sciences Tsinghua University, Beijing 100084, China.; ^4^ Institute of Analysis and Testing, Beijing Academy of Science and Technology, Beijing 100089, China.; ^5^ University of Chinese Academy of Sciences, Beijing 101408, China.

## Abstract

Biocatalytic regulation of dopamine signals paves an effective and biocompatible way to modulate dopaminergic functions and disorders. Here, we report the remolding of bacterial laccase, catalyzing conversions of dopamine and O_2_ to *o*-quinone and H_2_O, into a biocatalytic neuromodulator by reactive oxygen species–free dopamine catabolism. Given the poor activity of native laccase in the physiological context because of OH^−^ inhibition of its geometrically constrained O_2_-reducing center, we designed a highly dynamic Ru-Cu binuclear center to counteract the inhibition effect. Structural and computational investigations unravel a self-adaptive catalytic mechanism by reversive Ru-Cu active site reconfiguration that lowers the kinetic barriers for O_2_-to-H_2_O conversion in neutral solution. The remolded laccase exhibits substantial enhancement of physiological activity (up to one order of magnitude) and improved catecholamine substrate specificity, enabling whole-cell down-regulation of vesicular dopamine and extracellular erasure of evoked dopamine signals in intact brains. Our work elucidates a picture of artificial metalloenzymes for neuromodulation through a rationalized neurotransmitter metabolism pathway.

## Teaser

An artificial metalloenzyme catalyzing ROS-free dopamine oxidation is designed for multilevel neuromodulation.

## INTRODUCTION

Dopamine—often termed the “pleasure neurotransmitter” or “reward molecule”—plays essential roles in building the motor and cognitive functions of the brain. Abnormalities in dopamine concentrations in neurons or cerebrospinal fluids that lead to dysregulation of dopaminergic functions are the hallmark of numerous neurological and psychiatric disorders. Diminished dopamine levels are often associated with neurodegenerative diseases like Parkinson’s disease (*1*), while excessive dopamine signaling also causes motor dysfunctions, neuron degeneration, and psychiatric disorders (*2*–*4*). Current therapeutic strategies largely focus on compensating for synaptic dopamine deficiency by enhancing intracellular dopamine synthesis or inhibiting dopamine reuptake or metabolism (*5*–*10*). In sharp contrast, effective methods for treating abnormally intensified dopamine signals are scarce. Postsynaptic dopamine receptor antagonists are the available molecular therapeutics for dopamine-mediated overexcitation under psychiatric conditions like schizophrenia, although they can cause severe side effects and unpredictable risks (*11*). Hence, there is an urgent need for alternative approaches to modulate dopamine signals.

Enzymes are the natural tools to regulate neurotransmitter dynamics with high efficiency, specificity, and biosafety. Monoamine oxidase (MAO), an outer mitochondrial membrane–anchored enzyme, is crucial for dopamine catabolism by oxidative deamination (*12*). While, in principle, being able to serve as a biocatalytic modulator of dopamine neurotransmission, natural MAO lacks substrate specificity and promiscuously metabolizes serotonin, tyrosine, and phenylalanine, potentially interfering with other neurotransmitter systems. Moreover, the function of MAO is dependent on its cellular localization. When tethered to mitochondria, MAO catalyzes dopamine oxidation and transfers electrons to the membrane respiratory chain (*13*). Genetic removal of the membrane anchor can free the catalytically active domain of MAO into the cytosol, where dopamine oxidation is coupled to two-electron O_2_ reduction by an inherently bound flavin adenine dinucleotide, producing destructive reactive oxygen species (ROS) like H_2_O_2_ and exacerbating oxidative stress (*13*). Therefore, designing an artificial metabolic tool for dopamine with improved selectivity and biocompatibility is of great significance, albeit challenging.

**Fig. 1.**
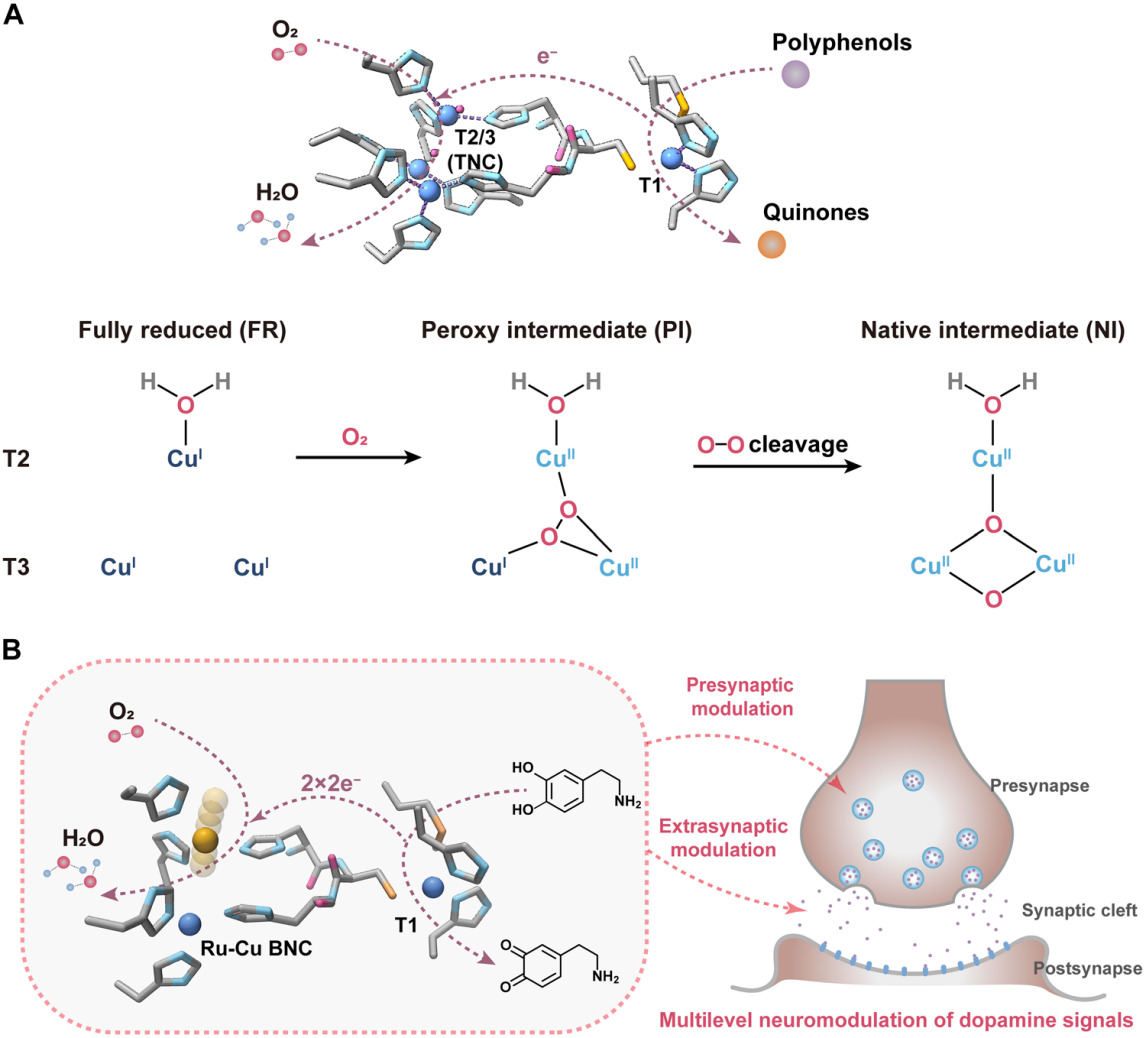
Schematic illustrations of laccase-based biocatalytic modulation of dopamine. (**A**) Enzymatic polyphenol oxidation catalyzed by natural laccase (PDB: 3CG8). The T2/T3 Cu-TNC forms an O_2_-bridged *m*_3_-1,1,2 peroxyl intermediate (**PI**) to break the O─O bond and transforms to a native intermediate (**NI**) carrying an O(H) bridge over T3 Cu sites, which is prone to inhibition by OH^−^ when pH increases. (**B**) ROS-free dopamine oxidation catalyzed by the remolded laccase for presynaptic and extrasynaptic down-regulation of dopamine signals. The designed Ru-Cu BNC in place of the Cu-TNC features an ultradynamic Ru site to resist the hydroxide inhibition effect and enhance physiological activity.

Laccase, a class of multicopper oxidases sourced from plants, fungi, and bacteria (*14*), presents a promising avenue to nonnatural ROS-free dopamine metabolism. The catalytic advantage stems from a type 1 (T1) mononuclear Cu center to oxidize polyphenols (e.g., catecholamine) and a unique type 2/type 3 (T2/T3) trinuclear Cu cluster (Cu TNC) to exclusively reduce O_2_ to water, thereby avoiding ROS generation (Fig. 1A). However, laccases are generally intolerant to the physiological environments, in which they suffer from inhibition of the Cu TNC by hydroxide anions (OH^−^) and exhibit poor activities at pH > 6 (*15*–*17*). In this regard, boosting laccase performance under close-to-neutral conditions holds the key to unlocking its neuromodulative applications in the real neurological context.

Here, we report an artificial laccase for physiological dopamine catabolism (Fig. 1B). We exploited bacterial small laccase (SLAC) (*18*) to remold its T2/T3 Cu TNC into a dynamically cooperated Ru-Cu binuclear center (BNC), which promotes four-electron O_2_ reduction with turnover frequency (TOF) remarkably exceeding that of Cu TNC in a pH-universal manner by up to one order of magnitude. Mechanistic investigations unraveled the self-adaptive formation of a transiently coupled Ru(─OO)─N─Cu intermediate to accelerate O_2_ activation and proton-coupled reductive O─O cleavage under close-to-neutral conditions. Compared to the native counterpart, the remolded SLAC exhibited boosted oxidase activity with improved substrate specificity to catecholamine. Using single-vesicle electrochemical sensing, we showed its performance in whole-cell biocatalysis of presynaptic dopamine transformation, reducing both vesicular storage and release of dopamine. Furthermore, with the fast-scan cyclic voltammetry (FSCV) technique, we illustrated that the artificial enzyme applied into the extrasynaptic space of living mouse brain can effectively erase stimulated dopamine transients, demonstrating its in vivo applicability for intervening with abnormally potentiated dopaminergic functions.

## RESULTS

### Rationale for chemical engineering of laccase

Among bioavailable metal sources, Cu holds a dominant position in oxygen bioactivation because of high d-electron density and redox potentials (*19*, *20*). This establishes the T2/T3 Cu TNC as a natural benchmark selectively converting O_2_ to H_2_O at the lowest activation energies known (*21*–*25*). The biocatalytic turnover is governed by the kinetics of reductive cleavage of an O_2_-bridged μ_3_-1,1,2 peroxyl intermediate (**PI**) to form a native intermediate (**NI**) with a μ_3_-oxo in the center of the TNC and a μ_2_-OH bridging the T3 Cu (*20*). Under physiologically relevant conditions, an increase in OH^−^ concentration leads to strong binding of an OH^−^ anion over T3 Cu sites, retarding both **NI** formation and its decay back to the resting fully reduced (**FR**) state. The hydroxide inhibition of O_2_ reduction arises from the stable geometry of Cu TNC immobilized in the native laccase binding pocket, where closely spaced and spatially restrained T3 Cu sites are easily coupled through O_2_ or OH^−^. Therefore, our strategy for redesigning laccase toward enhanced physiological activity involves relaxing the geometric constraints on the O_2_-reducing sites.

In nature, flexible metalloprotein multimers can adopt different conformations depending on the coordination strengths and geometric preferences of metal ions, enabling multisite coupling to regulate metal-binding states (*26*, *27*). Inspired by this phenomenon, we propose a protein-protein interface–assisted chemical approach to construct a dynamically reconfigurable O_2_-reducing site that mitigates the hydroxide inhibition effect. Our design is based on three key criteria: the laccase template, the choice of metal cations, and the sequence of metal incorporation. For this purpose, we selected SLAC for further remolding because of its ability to dissociate into monomers in the *apo* state while dimerizing upon metal binding (*18*, *28*). The monomeric *apo*-SLAC features a constrained T1 site for selective Cu binding and two distal surface regions rich in histidine residues that can chelate diverse metal cations, leading to metal-induced formation of a flexible interfacial cleft replete of coordinable functionalities (fig. S1).

We chose Ru^3+^ and Cu^2+^ to sequentially construct the first and second metal sites at the interface for two main reasons. First, compared to Cu(II/I), Ru(III/II) exhibits higher d-electron density and binding affinity to oxygen-containing species, which is expected to enhance O_2_ activation. Second, Ru^3+^ has a larger ionic radius (0.81 Å) than Cu^2+^ (0.72 Å) and a longer equilibrium length of Ru(III)─N_imidazole_ bond with smaller equilibrium force constant than that of the Cu(II)─N_imidazole_ bond [generated with Multiwfn (*29*) and Sobtop (*30*)] (table S1). According to the Irving-Williams series, Cu^2+^ is the most protein-affinitive bioavailable metal cation (*31*); thus, its localization at the interface is likely to induce local reorganization because of its dominant binding strength. Consequently, the Ru site may shift from its prebound equilibrium state to a nonequilibrium dynamic state, allowing it to actively pursue the most kinetically favorable pathway even under unfavorable conditions.

We conducted a brief 5-ns molecular dynamics (MD) simulation to evaluate the behaviors of free Ru^3+^ and Cu^2+^ at the SLAC dimer interface without assigning specific bonds (fig. S2). The Cu atom is poised to a relatively static position by five surrounding histidine residues [His^102^ (H102), H104, H156, H234, and H289]. In contrast, the Ru atom shows greater spatial movement under interactions with only two histidine residues (H158 and H287) and two carboxylates [Asp^113^ (D113) and Glu^163^ (E163)]. We measured the distances between the metal atoms and nearby coordinable residues every 2 ps in simulated trajectories (fig. S3). Distributions of Ru-N_His_/O_Asp_/O_Glu_ distances are more dispersive (*R* values: 0.67 to 4.43 Å) than those of Cu-N_His_ distances (*R* values: 0.44 to 0.60 Å), suggesting that the Ru-centered motif may adopt a more dynamic conformation.

### Construction and elucidation of a dynamic Ru-Cu BNC

**Fig. 2.**
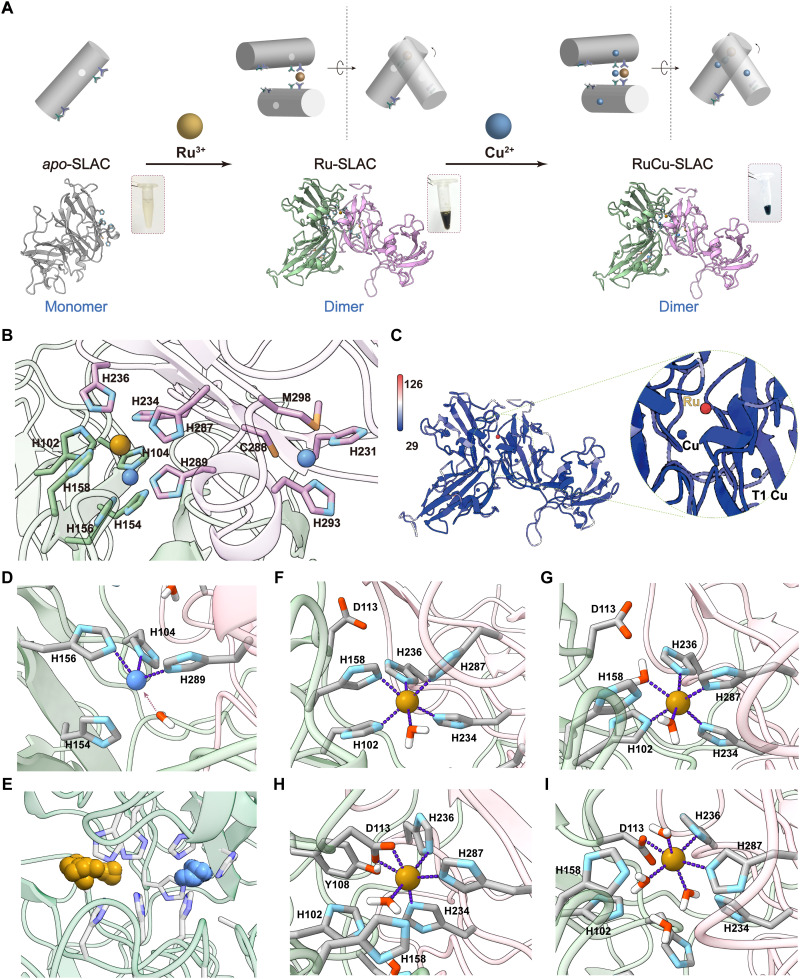
Laccase remolding and structural elucidation. (**A**) Schematic illustration of a two-step construction of the histidine-coordinated Ru-Cu BNC at the SLAC dimer interface and mononuclear Cu at the T1 site. (**B**) Crystallographic elucidation of the Ru-Cu BNC at the dimer interface, connected to mononuclear Cu at the T1 site by H287/C288/H289. (**C**) Global *B* factor mapping of all RuCu-SLAC atoms. Inset: zoom-in view of the interface-confined Ru-Cu BNC and T1 Cu in *B* factor coloring. Color scale bar, *B* factor amplitude. (**D**) Simulated interfacial Cu^2+^ center in tricoordinated geometry or tetracoordinated geometry if the water molecule is taken as a weak ligand. (**E**) Simulated atomic occupancy of single-bonded Cu^2+^ and Ru^3+^ at the SLAC dimer interface. (**F** to **I**) MD-identified structures of Ru^3+^-centered motif **1** (F), motif **2** (G), motif **3** (H), and motif **4** (I) out of 10-ns trajectories.

We set out to construct the Ru-Cu BNC through a two-step sequential metal incorporation scheme (Fig. 2A). We first incubated *apo*-SLAC templates (2 mg ml^−1^) with RuCl_3_‧3H_2_O (1 mM) in 50 mM tris buffer (pH 8.0) at 4°C overnight and obtained a red-brownish purified protein that displayed UV-visible (UV-vis) absorbance bands at 350 and 430 nm (fig. S4), a sign of successful implementation of Ru^3+^ onto protein scaffolds (Ru-SLAC). Protein gel electrophoresis and gel filtration show that the monomeric *apo*-SLAC bands mostly turned to dimeric after Ru incubation (fig. S5), indicating that Ru^3+^ coordinates to the surface-exposed histidine sites on two *apo*-SLAC monomers and mediates the protein dimerization. A small portion of proteins underwent trimerization (molecular weight: ~110 kDa), which may form a threefold symmetric trimer through Ru-mediated “head-to-tail” assembly as observed with native SLAC (fig. S6) (*18*). Larger protein oligomers were not observed under conditions of interest, ruling out random metal binding–induced protein aggregation. Then, we conducted a secondary incubation of Ru-SLAC dimers with CuCl_2_ at different stoichiometric ratios and incubation time lengths. The dark-green purified protein gives intense absorbance at 600 nm assigned to the ligand-metal charge transfer transition of T1 Cu(II) (*21*). Absorbance bands spanning 300 to 500 nm also rise with the increases in CuCl_2_ concentration and incubation time (fig. S4). Last, we adopted a stoichiometric ratio of 1:10 and 2-hour incubation to construct the artificial metalloenzyme (named as RuCu-SLAC) for investigation throughout the study. For comparisons, we reconstituted native SLAC by a one-step incubation of *apo*-SLAC templates with CuCl_2_ based on the reported method (*18*).

The RuCu-SLAC homodimer accommodates one Ru and three Cu atoms as suggested by inductively coupled plasma mass spectrometry (ICP-MS) (table S2). The protein crystal structure (2.1 Å) supports the ICP-MS result and clarifies the exact positioning of a Ru-Cu BNC within the binding interfacial cleft and two mononuclear T1 Cu sites on subunits (Fig. 2B). H231, Cys^288^ (C288), and H293 constitute the highly conservative primary sphere of T1 Cu. The entire Ru-Cu BNC is surrounded by nine histidine residues across the interface. Cu^2+^ coordinates to H289 on subunit A and H104/H156 on subunit B at Cu(II)─N_His_ bond lengths of 2.0, 1.9, and 2.0 Å. Ru^3+^ is only tethered by H287 on subunit A and H158 on subunit B (Ru─N_His_ bond lengths: 2.5 and 2.2 Å). In the single-crystal phase, Ru-Cu spacing is 4.7 Å, too far for direct coupling. No bridging atoms (i.e., oxygen atoms from H_2_O or O_2_) are observed.

To assess the dynamic nature of the remolded active site, we globally mapped *B* factors (or Debye-Waller factors) of RuCu-SLAC and found that a Ru atom has a remarkably larger value (112.65 Å^2^) than Cu atoms (36.83 Å^2^ for interfacial Cu and 38.03 Å^2^ for T1 Cu) and the rest of protein scaffold (Fig. 2C). As an important sign of electron density dispersion and thermal stability, a *B* factor increase reflects uncertainty in the assigned location of the Ru atom, correlated with conformational transitions from the most stable equilibrium state to metastable or nonequilibrium states. Ru in a crystallized sample has a low occupation ratio of only 0.30, and interfacial Cu also displays a reduced occupation ratio of 0.70.

We next sought to simulate dynamics of the Ru-Cu BNC in solvated proteins, starting from crystallographic atom coordinates and assigning real bonds of Ru─N_H287_ and Cu─N_H289_. Along with equilibration of protein backbone atoms, an interfacial Cu–centered motif (with H104/156/289) is equilibrized to a distorted tetrahedral configuration, transitioned from the crystalline near-planar triagonal configuration upon approaching of H154 side chain and one water molecule toward Cu (within 3.0 Å) from the outer side (Fig. 2D). In sharp contrast, the Ru atom swings back and forth in the cleft and forms dynamic connectivity with proximal functionalities (Fig. 2E). We analyzed clusters of Ru-centered motif structures and identified four distinct hexacoordinated configurations out of 10-ns trajectories (Fig. 2, F to I). Motif **1** containing five histidine residues (H102/158/234/236/287) and one water molecule existed in the beginning stage (~1 ns) of MD production run. As H158 drifted away, another water molecule bound Ru to satisfy a hexacoordination (motif **2**). Afterward, a new aspartate residue (D113) appeared in proximity of Ru (<2.5 Å)—also observed in nonbonded MD simulation—and drove the leaving of H102 and one water ligand (motif **3**). At *t* = ~5 ns, the primary coordination sphere consisted of only two histidine residues (H236/287), three water ligands, and D113, forming motif **4** with the longest lifetime. The dynamic coordination network of Ru well agrees with its high *B* factor value and low occupation ratio determined by electron density mapping.

To clarify the electronic structure and chemical environment of the Ru center, we performed x-ray absorption near-edge structure (XANES) and extended x-ray absorption fine structure (EXAFS) analysis. We collected the Ru K-edge XANES spectrum of RuCu-SLAC using Ru foil and RuO_2_ as references (fig. S7A). The absorption edge shifts close to that of RuO_2_, assigning a high valence state to the Ru center (~+3). The Fourier-transformed *k*^2^-weighted EXAFS spectrum of the *R* space for Ru exhibits three major scattering peaks of Ru─N or Ru─O coordination (fig. S7B). Stepping further, we assumed a set of simplified Ru coordination configurations for the least-square EXAFS curve fitting including the *R* space. The best-fit model suggests the presence of at least two histidine residues and one water molecule in the primary coordination sphere, in which Ru─O_water_ coordination contributes to the EXAFS peak at 1.4 Å, while the two Ru─N_His_ coordination bonds scatter at 1.8 and 2.2 Å (fig. S7C). The fitting result stands in line with MD prediction and verifies the presence of aqua ligands at the Ru center in the aqueous phase. Minor EXAFS peaks among 2.5 and 4.0 Å may reflect weak interactions of Ru with histidine residues in distances too long for reasonable bonds. These side-chain rotamers could make up the secondary coordination sphere to regulate metal activity.

### Catalytic activity, selectivity, and kinetics of remolded laccase

As shown in Fig. 1, laccase operates through a bipolar catalytic scheme: Dopamine undergoes a two-electron oxidation by the T1 Cu(II) site to generate *o*-quinones, and the abstracted electrons are quickly transferred to the other site to reduce O_2_. To verify that the remolded laccase retains the mechanism, we characterized the products of half-cycle reactions. Using dopamine as a substrate, we detected a strong absorption peak around 480 nm with native SLAC or RuCu-SLAC (fig. S8), indicating that both enzymes catalyze the same dopamine transformation. The *o*-quinone product of RuCu-SLAC catalysis was further validated by nuclear magnetic resonance spectroscopy (fig. S9). For the reductive half-cycle reaction, we conducted an electrochemical analysis to demonstrate negligible H_2_O_2_ production during O_2_ reduction with the Ru-Cu BNC, with an electron transfer number of 3.9 (described in Materials and Methods and fig. S10). This indicates that a total of four electrons, provided by two dopamine molecules, is needed to convert O_2_ to H_2_O in a single enzymatic turnover.

**Fig. 3.**
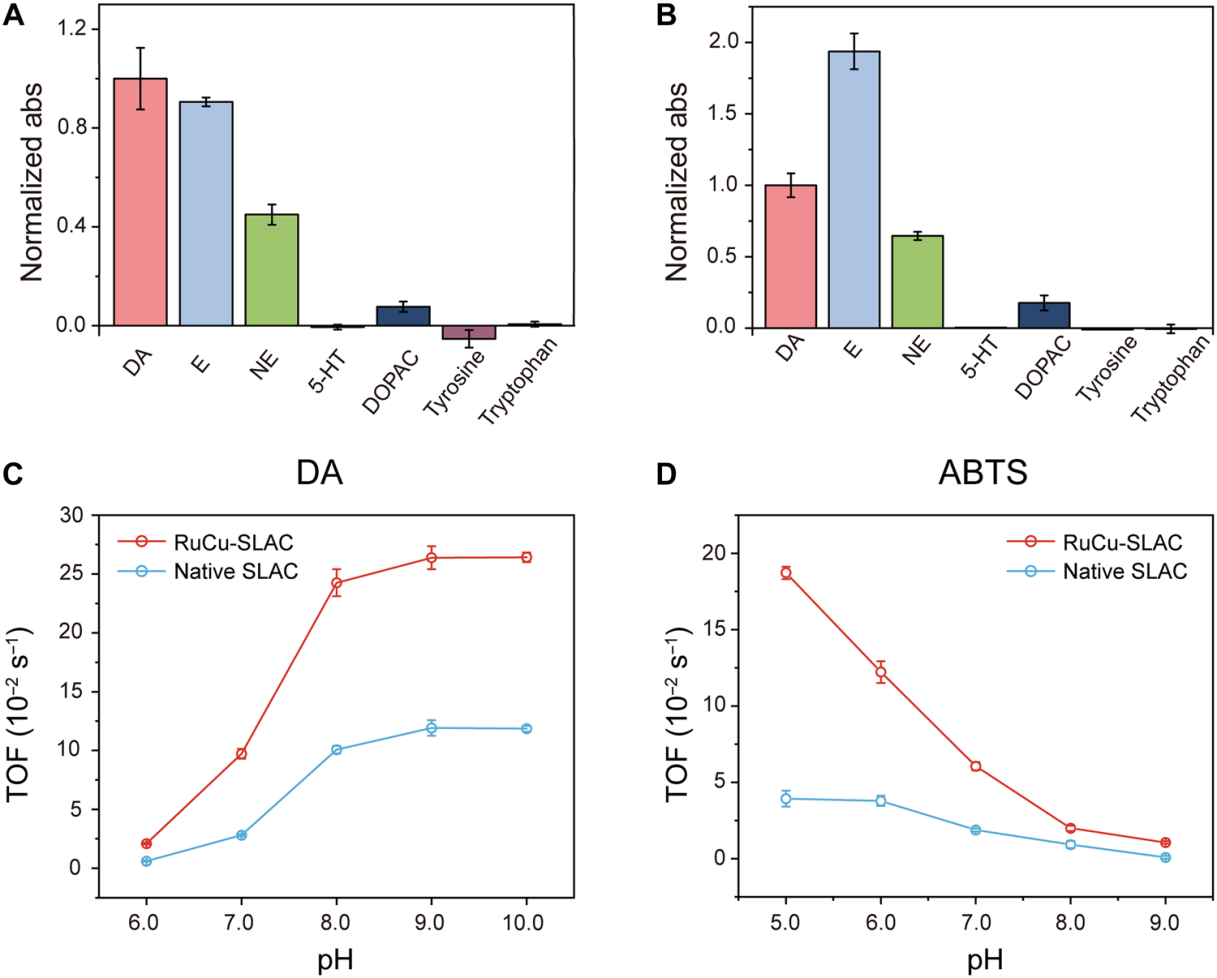
Comparative investigations of oxidase performance. (**A** and **B**) Relative catalytic activities of RuCu-SLAC (A) and native SLAC (B) to monoamines and dopamine metabolites, represented by normalized absorbance (abs) changes of oxidation products. DA, dopamine; E, epinephrine; NE, norepinephrine; 5-HT, serotonin; DOPAC, dihydroxyphenyl acetic acid. (**C** and **D**) TOF values of RuCu-SLAC and native SLAC for the catalysis of dopamine oxidation (C) and ABTS oxidation (D) at pH 6.0 to 9.0.

Next, we examined the substrate specificity of RuCu-SLAC. Biocatalytic activities toward monoamines were assessed by spectroscopic scanning of respective product formation (fig. S11). As shown in Fig. 3A, RuCu-SLAC exhibits oxidase activities toward dopamine, epinephrine, and norepinephrine, all sharing bisphenol and amine moieties. For other coexisting aromatic monoamines like serotonin, tyrosine, and tryptophan, RuCu-SLAC showed no biocatalytic activities. Such a tendency of substrate-specific activities is similar to that obtained with native SLAC (Fig. 3B), implying that the T1 Cu site determining the substate affinity was merely unaffected by enzyme remolding on the other site. By superimposing RuCu-SLAC onto native SLAC, we showed high-degree overlapping T1 sites, ruling out the possibility that Ru-Cu BNC incorporation may induce distant conformational changes to affect substrate oxidation (fig. S12).

Compared to the native SLAC, RuCu-SLAC showed substantial enhancement in dopamine-to-quinone conversion at pH 6.0 to 9.0 (fig. S13). Michaelis-Menten constants and *K*_cat_ (turnover number) of both enzymes with a dopamine substrate in the physiological pH range are summarized in table S3, indicating a monotonic enhancement of catalytic kinetics of RuCu-SLAC with pH increasing from 6.0 to 8.0. Given the common halide and thiol inhibition on laccase performances, we also monitored enzymatic reactions in the presence of chlorides or cysteines, both being abundant in the brain. RuCu-SLAC and native SLAC exhibited comparable resistance to a low-to-high level of NaCl (0 to 500 mM) without obvious activity loss (fig. S14). Introducing reductive cysteines into the assay cocktail resulted in delays of quinone production. Increasing the cysteine concentration led to prolonged lag time but did not affect the quinone production rate (fig. S15). Nevertheless, the cysteine-induced lag time is much shorter with RuCu-SLAC and suggests faster enzyme kinetics.

Excluding the contribution of dopamine self-oxidation, we quantitatively evaluated the single-enzyme catalysis of dopamine metabolism by TOF, which is the universal kinetic parameter for assessing the intrinsic activity of a biological or artificial catalyst (*32*). As shown in Fig. 3C, TOF values of RuCu-SLAC or native SLAC increase with pH shift to the alkaline regime (6.0 to 9.0), given that the half-cycle dopamine oxidation is pH-dependent and facilitated at higher pH levels. Under close-to-neutral conditions, RuCu-SLAC exhibits remarkably higher TOFs of 0.02 s^−1^ at pH 6.0, 0.10 s^−1^ at pH 7.0, and 0.24 s^−1^ at pH 8.0, enhanced by 3.0-, 2.6-, and 1.4-fold as compared to native SLAC, respectively. Given that O_2_ reduction is retarded in a close-to-neutral solution, there is a tradeoff between reaction kinetics at the T1 Cu site and the interfacial site. To validate that the physiological activity enhancement of remolded laccase was mainly credited to promoted O_2_ reduction with the Ru-Cu BNC, we further chose 2,2′-azinobis-(3-ethylbenzthiazoline-6-sulfonate) (ABTS) as the electron donor, which loses one electron at the T1 site to yield a cation radical product (ABTS^•+^) strongly absorbing at 420 and 650 nm (fig. S16A). This step is fast and pH-independent, so the complete reaction rate is determined by reaction kinetics of the Ru-Cu BNC or T2/T3 Cu TNC. Again, we show that RuCu-SLAC catalyzes ABTS oxidation more efficiently than native SLAC in a wide pH range (fig. S16B), with TOF magnification (TOF_RuCu_/TOF_native_) being two- to threefold at pH 6.0 to 8.0 (Fig. 3D). Notably, the Ru-Cu BNC brings about one order of magnitude enhancement (11.5-fold) of TOF (1.17 × 10^−2^ s^−1^) at pH 9.0, where native SLAC is nearly inactive (0.09 × 10^−2^ s^−1^). Even under native SLAC-favored acidic condition (pH 5.0), RuCu-SLAC exhibited TOF magnification of almost 4-fold. Collectively, we demonstrate that the kinetic advantage of RuCu-SLAC over native SLAC is pH-universal and remarkable.

### Structural origin of boosted physiological activity

Given the coupled catalytic mechanism, TOF is determined by the T1 oxidation rate, intramolecular electron transfer (IET) rate, and interfacial O_2_ reduction rate. As the contribution of T1 Cu to kinetic enhancement is trivial, we considered whether the IET distance changed to affect synergy between the T1 site and the interfacial site, separated by about 12.8 Å in native SLAC. Ru-Cu_T1_ distances and Cu_BNC_-Cu_T1_ distances extracted from MD trajectories of RuCu-SLAC are both averaged at 13.4 Å with narrow variations (fig. S17). The increase in IET distance (0.6 Å) in RuCu-SLAC is so small that its impact on synergistic catalysis should be negligible. In this regard, the boosted physiological activity of the remolded laccase can be credited to the promoted catalysis of O_2_ reduction, which is dictated by the structure of the Ru-Cu BNC.

**Fig. 4.**
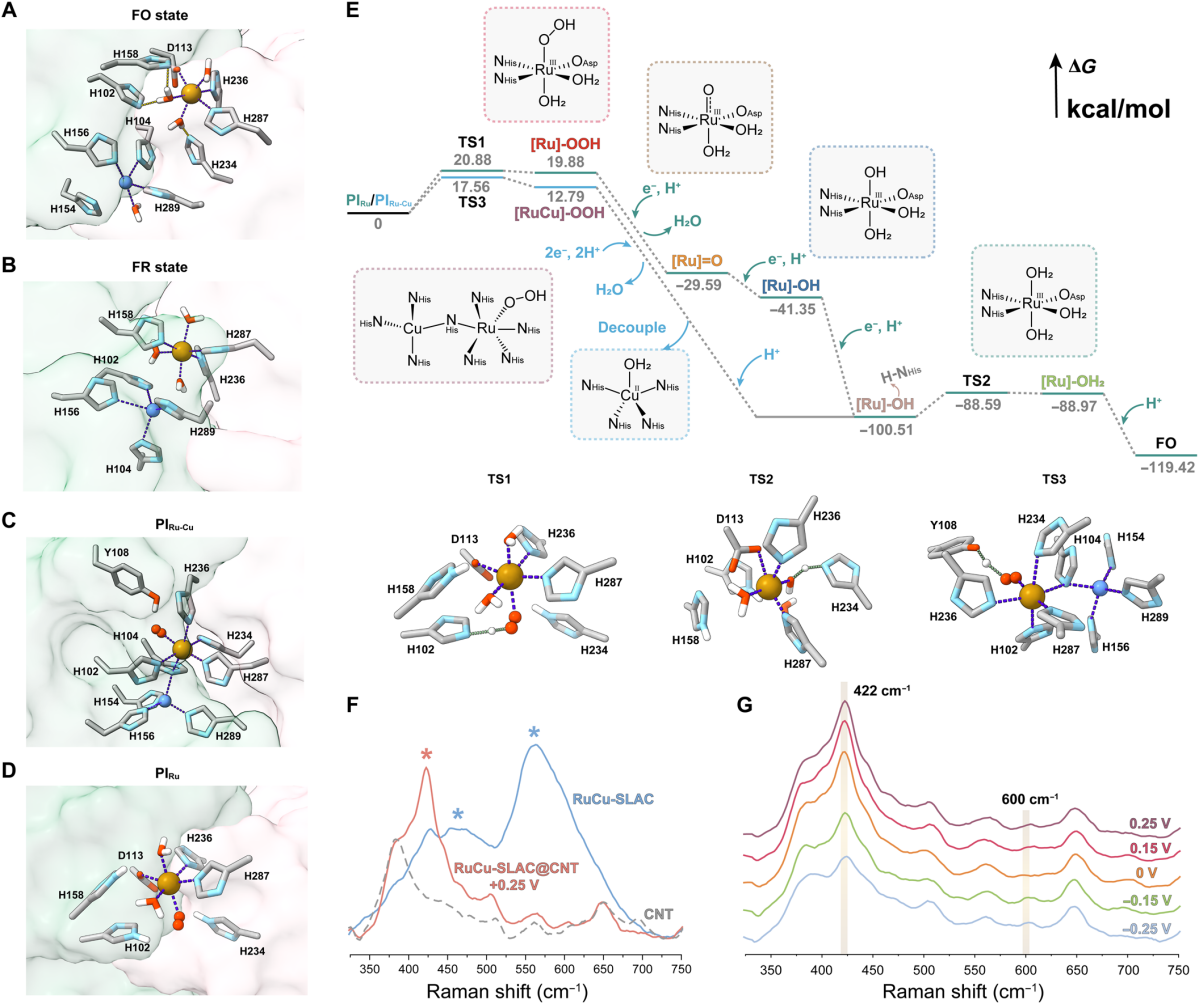
Mechanism for Ru-Cu BNC–catalyzed O_2_ reduction. (**A** and **B**) Simulated structural transition of Ru(III)-Cu(II) (**FO** state) (A) to Ru(II)-Cu(I) (**FR** state) (B) with neutralized histidine residues in the absence of O_2_. (**C** and **D**) Simulated structures for the coupled [Ru(O_2_)─N─Cu]^3+^ intermediate (**PI**_**Ru-Cu**_) (C) and the noncoupled [Ru─O_2_]^2+^ intermediate (**PI**_**Ru**_) (D). (**E**) Proposed O_2_ reduction pathways at the Ru-Cu BNC with calculated free energy changes. Transition-state structures deduced from cluster models of **PI**_**Ru**_ and **PI**_**Ru-Cu**_ are illustrated. Proton abstraction at transition states is indicated by dashed green lines. (**F**) Overlay of experimental Raman spectra of RuCu-SLAC at the resting state, RuCu-SLAC immobilized on the CNT-modified electrode biased at +0.25 V (versus Ag/AgCl), and pristine CNT background. Characteristic peaks of the catalyst (not detected on the background spectrum) are marked by asterisks. (**G**) Bias potential–dependent changes in Raman signals of Ru─O stretching in coupled **PI**_**Ru-Cu**_ and decoupled **Ru(II)-OH** intermediates.

The tetracoordinated Cu(II) site and motif **4**-like Ru(III) site are separated by 5.6 to 10.4 Å in the majority of simulated ensemble with neutralized surrounding histidine residues in the absence of O_2_ at the fully oxidized (**FO**) resting state (Fig. 4A and fig. S18). Water ligands of the Ru(III) site may form hydrogen bonds with imidazole side chains of H102, H158, and H234. Once fully reduced (**FR**), bond force constants of Ru(II) and Cu(I) to histidine residues are increased and equilibrium bond lengths are decreased (table S1). Reinforced coordination shortens the Ru-Cu distance to 3.7 to 8.8 Å (fig. S18). In the dominant **FR** conformation (Fig. 4B), the Cu(I) site regains the near-planar tricoordinated configuration by moving toward H102 and leaving H156 in its axial direction at 3.0 Å. Meanwhile, the Ru(II) center adopts a new motif configuration consisting of three histidine residues (H158/236/287) and three water ligands. At either state, two metal centers are not coupled, so it is necessary to clarify the O_2_ binding site. We used density functional theory (DFT) to compute binding energies (BEs) of O_2_ to the Ru(II) or Cu(I) site. Modeled by simplified metal-ligand complexes (fig. S19), electronic energies for O_2_ binding with Ru(II) in various coordination configurations are very negative (−30 to −100 kcal/mol), 8 to 25 times of that for Cu(I)─O_2_ (less than −5 kcal/mol). The Ru(II) site exerts much higher O_2_ affinity, thus assigned as the O_2_ binding site.

According to MD simulations, the oxyRu(II) site induces notable local reconfiguration to form the catalytically relevant peroxyl intermediate of [Ru(O_2_)─N─Cu]^3+^ (**PI**_**Ru-Cu**_; Fig. 4C), in which the neutral imidazole ring of H104 vertically inserts between Ru and Cu to form a dynamic bridge at ~2.5 Å through d-π conjugation (fig. S20). A tyrosine residue [Tyr^108^ (Y108)] appears nearby (<3.0 Å) because of probable attractive force between the phenolic hydrogen and Ru-bound O_2_, the latter being oriented to a direction perpendicular to the linear Ru-N-Cu axis, unlikely forming the ─O─O─ bridge over the Ru-Cu BNC. A noteworthy factor for successful formation of **PI**_**Ru-Cu**_ is deprotonation of H158 and H234 side chains to allow ligand exchange on Ru. We calculated p*K*_a_ (where *K*_a_ is the acid dissociation constant) values of ionizable residues in RuCu-SLAC and obtained the side-chain protonation tendency of H234 > H158 > H156 > H289 > H102 > H236 > H104 > H287 > H154 (table S4). Apparently, H234 (p*K*_a_ of 8.6) and H158 (p*K*_a_ of 8.2) are the most basic of the nine residues, and they tend to be protonated at pH < 8.0. In acidic media (pH 6.0 to 5.0), Ru(II)-H158 interaction at the **FR** state and Ru(II)-H234 interaction at the O_2_-bound state would be weakened, disfavoring sequential transitions to **PI**_**Ru-Cu**_. For this situation, we proposed a noncoupled mononuclear [Ru─O_2_]^2+^ intermediate model (**PI**_**Ru**_; Fig. 4D) built on the motif **4** structure by replacing one water ligand with O_2_. After restricted geometric optimization with protonated H234, H158, and H102, the bound O_2_ points to the middle of H102 and H234, a beneficial orientation for proton abstraction from both sides.

To gain mechanistic insights, we conducted cluster-model DFT calculations of elementary conversions from peroxyl intermediates (Fig. 4E). For acidic catalysis beginning with **PI**_**Ru**_, we deduced a mononuclear pathway involving four intermediates: [Ru]-OOH, [Ru]═O, [Ru]-OH, and [Ru]-OH_2_. O_2_ activation accompanied with proton abstraction from H102 (**TS1**) has a low free energy (Δ*G*) barrier of 20.88 kcal/mol, followed by proton-coupled electron transfer that renders quick O─O cleavage. A strongly basic [Ru]═O intermediate is easily protonated, while [Ru]-OH goes through another transition state of proton abstraction from H234 (**TS2**) at an even lower Δ*G* of 11.92 kcal/mol. Electrons for reducing Ru(III) intermediates can be donated by T1 Cu(I) at ~13 Å or by the interfacial Cu(I) site at ~8 Å at a higher transfer rate. Protons of nearby residues are donated by water molecules in the cleft. A few water molecules (H_2_O or H_3_O^+^) are found aligned between the Ru-Cu BNC, probably establishing a H-bonded highway for facile proton transport (fig. S21).

For neutral catalysis (pH 7.0 to 8.0) beginning with **PI**_**Ru-Cu**_, we postulated a binuclear-mononuclear hybrid pathway relying on dynamic Ru-Cu cooperation. Protonation of **PI**_**Ru-Cu**_ by Y108 (**TS3**) is facilitated by the side-on peroxide-Ru(II) at Δ*G* of 17.56 kcal/mol. The resulting [RuCu]-OOH intermediate displays polarized distribution of highest occupied molecular orbitals centered on Cu and lowest unoccupied molecular orbitals centered on Ru-peroxide (fig. S22). The bridging imidazole mediates orbital overlaps and backbonding interaction of π* electrons and σ* orbitals, eliciting strong antibonding to break the O─O bond. The presumable [RuCu]═O intermediate would be unstable because of the elongated O_Ru_-Y108 distance and decouple to [Ru]-OH upon subsequent proton-coupled electron transfers. The dynamic crossover is crucial for sustaining the turnover rate; otherwise, [RuCu]-OH must overcome a huge activation barrier (168.03 kcal/mol) to return to the resting state.

Experimental support of the DFT-suggested self-adaptive mechanism was obtained with in situ Raman spectroscopic characterizations. At the resting **FO** state, RuCu-SLAC intrinsically induces Raman shifts at 460 and 560 cm^−1^, assigned to Ru─O stretching and out-of-plane bending of the Ru-bound water ligand in the motif **4** configuration that give the calculated Raman signal at 464, 505, and 520 cm^−1^ (Fig. 4F and fig. S23). Given that capturing transient intermediates of a nonequilibrium catalyst in the solution phase is quite difficult, we turned to RuCu-SLAC electrocatalysis at carbon nanotube (CNT)–coated electrodes through direct electrochemical reduction of the T1 Cu site and then the Ru-Cu BNC (schematically illustrated in fig. S10). In the heterogeneous context, we were able to conduct operando analysis of intermediate transitions at the electrode-electrolyte interface. The RuCu-SLAC@CNTs–functionalized electrodes in the Tris-HCl–buffered electrolyte (pH 8.0) were polarized at a constant potential, and the corresponding Raman spectrum was sampled from the electrode surface. Starting at the redox potential of T1 Cu (0.25 V versus Ag/AgCl), we collected the spectroelectrochemical spectrum by shifting the bias potential negatively to −0.25 V at an increment of 50 mV. As shown in Fig. 4 (F and G), a sharp and intense Raman signal around 422 cm^−1^ emerges upon aerobic electrochemical reduction of RuCu-SLAC (not observed with pristine CNTs) and lasts in the low-overpotential region (0.25 to 0 V). We assigned it to the Ru─O stretching of **PI**_**Ru-Cu**_ with a calculated Raman signal at 343 cm^−1^ (fig. S24). In the meantime, the signals associated with mononuclear Ru─O almost disappear, implying the reconfiguration of the Ru-Cu BNC into the coupled form. As the bias potential shifted toward −0.25 V, we observed a decrease in the peak at 422 cm^−1^ and a gradual emergence of a small peak at 600 cm^−1^, which could be ascribed to high-overpotential accelerated transitions of **PI**_**Ru-Cu**_ to the mononuclear **Ru(II)-OH** intermediate (stretching around 595 cm^−1^ by calculation; fig. S25). It is worth mentioning that the relatively large deviations of calculated frequencies from the measured values are correlated with restricted optimization of cluster models with C, N, and metal atoms frozen to enable self-consistent field convergence at reasonable computational expenses. Local flexibility was manually reduced and cannot fully resemble the dynamic conditions. A general trend is that calculated Ru─O stretching frequencies are lower than the observed ones, because only O and H atoms were allowed to move in vibrations without contributions of backbone and metal atom displacements. We found that unrestricted optimization of **PI**_**Ru-Cu**_ would ultimately lead to a decoupled oxyRu site and Cu site, emphasizing again that the imidazole-coupled structure is not at equilibrium and apt to reconfigure when geometric constraints change with intermediate transitions. Through this self-adaptive mechanism, RuCu-SLAC attains enhanced physiological oxidase activity.

### Whole-cell modulation of presynaptic dopamine synthesis

**Fig. 5.**
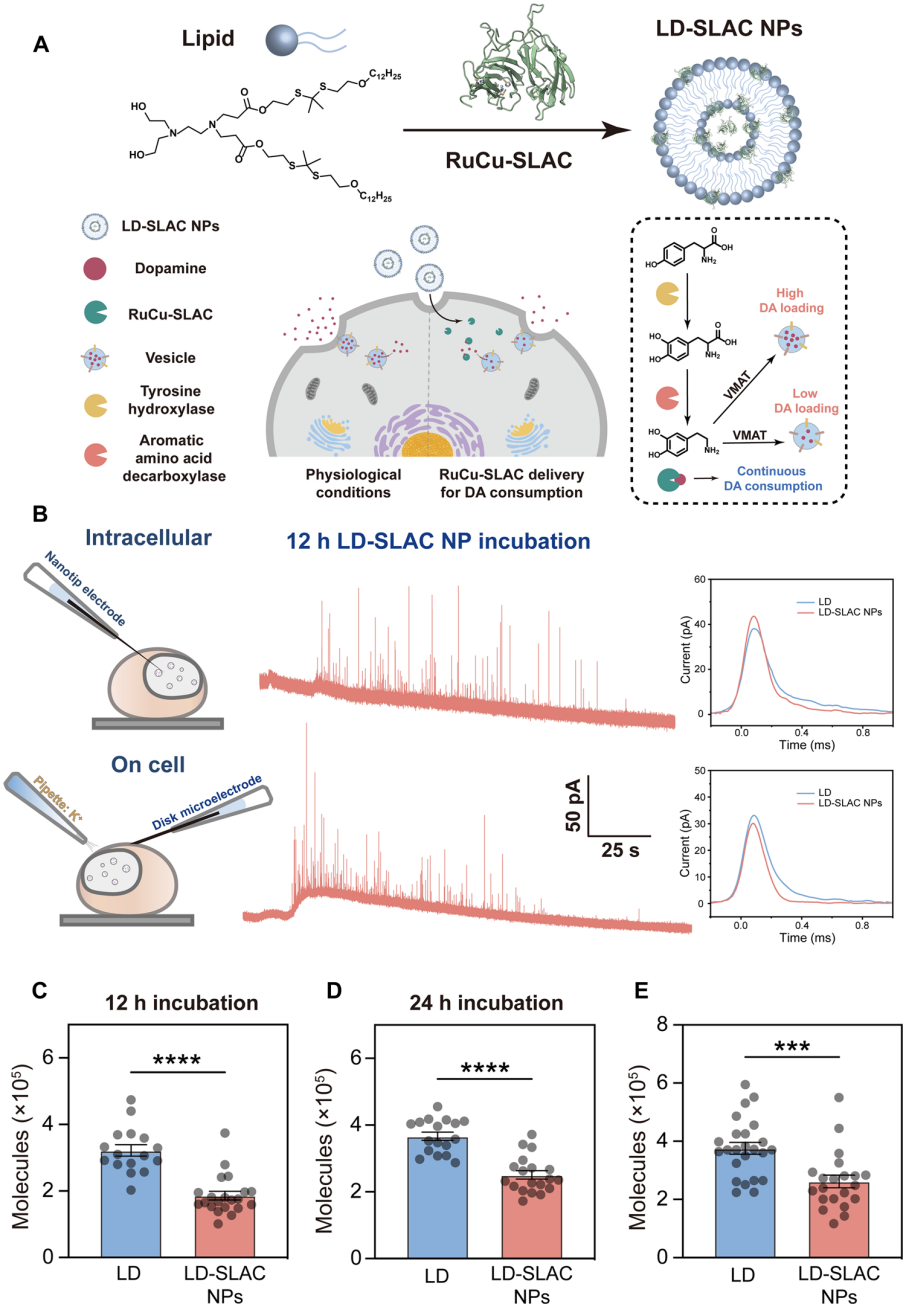
Whole-cell down-regulation of dopamine signals. (**A**) Schematic illustration of the intracellular delivery of RuCu-SLAC into the cytoplasm by ROS-cleavable liposomes for enzymatic consumption of dopamine. LD, lipid; VMAT, vesicle monoamine transporter. (**B**) Intracellular and on-cell measurements of vesicular dopamine storage and release content by single-vesicle electrochemistry. Typical current transients representing a single vesicle or single exocytotic event are shown. h, hour. (**C** and **D**) Measured total numbers of dopamine molecules stored in single cytosolic vesicles before and after RuCu-SLAC delivery for 12 hours (C) and 24 hours (D). Each data point represents the median value of all vesicles recorded inside a single cell. (**E**) Measured total numbers of dopamine molecules released in single-vesicle exocytosis before and after RuCu-SLAC delivery for 12 hours. Each data point represents the median value of all exocytotic events recorded on a single cell. ***P < 0.001; ****P < 0.0001.

As demonstrated, RuCu-SLAC offers the potential to alleviate dopamine-mediated symptoms and diseases by catalyzing dopamine conversion with improved physiological activity and selectivity. Assays of activity retention also validated enzyme stability despite the dynamic nature of the Ru-Cu BNC, evidenced by the finding that RuCu-SLAC maintained at least 80% of catalytic activity even after 48-hour incubation in physiological buffer at 4°C (fig. S26). Worth mentioning is the enzyme ability of intervening dopamine synthesis by catalyzing dihydroxyphenylalanine (or l-dopa) oxidation. In both ways, RuCu-SLAC may reduce vesicular packaging and the release of dopamine, which are the presynaptic steps for neurotransmission. The general workflow for whole-cell validation consists of two steps, cellular enzyme delivery and in situ quantification of biocatalytic effects. In the first step, we used a developed liposomal delivery strategy (*33*) that allowed the coassembly of ROS-cleavable amphiphilic lipids and RuCu-SLAC into protein-encapsulated nanoparticles, entering into cells through endocytosis and triggered by intrinsic ROS to release RuCu-SLAC into the cytoplasm (Fig. 5A). Using fluorescein isothiocyanate (FITC)–labeled RuCu-SLAC, we confirmed that a 12-hour cell incubation with the lipid-protein nanoparticles (LD-SLAC NPs) can achieve a cytosolic delivery efficiency of more than 60% on dopaminergic PC-12 cells (fig. S27).

Internalized RuCu-SLAC is supposed to catalytically consume l-dopa to cut down its decarboxylation to dopamine or directly consume dopamine before its transport into vesicles. Thus, in the second step, we used advanced single-cell/vesicle electrochemical analysis to quantify downstream dopamine amounts in situ. Fig. 5B depicts the measurement setups and typical outcomes for individually counting dopamine molecules packed in intracellular vesicles and released from vesicle exocytosis, as detailed in Materials and Methods. As shown in Fig. 5C, the average vesicle storage of dopamine in PC-12 cells treated with LD-SLAC NPs for 12 hours decreases to below 70% of that in the control group (i.e., PC-12 cells treated with unloaded liposomes). Prolonging the incubation length to 24 hours did not further reduce the dopamine storage but maintained it at the 70% level (Fig. 5D), suggesting steady and persistent functioning of internalized RuCu-SLAC for cytosolic dopamine catabolism to strike a new equilibrium with continuous dopamine synthesis. Correspondingly, vesicles of LD-SLAC NP–treated cells release fewer dopamine molecules during potassium-triggered exocytosis, statistically 60% of the control level (Fig. 5E). The intracellular dopamine level can be restored at 48 hours (fig. S28) when the delivered RuCu-SLAC was completely cleared from the cytosol (fig. S29), so we can estimate that the intracellular half-life of RuCu-SLAC after delivery is within 24 to 48 hours. By contrast, cells after the delivery of native SLAC displayed insignificant changes in the exocytotic dopamine release as compared to the control group or cells transfected with *apo*-SLAC (fig. S30). Despite in vitro catalytic activities, native SLAC appeared inactive for dopamine catabolism in the cytosolic environment. These single-vesicle/cell investigations provide a direct proof of RuCu-SLAC for short-term modulation of dopamine concentration in the biological context.

### In vivo modulation of dopamine signals in mouse brains

**Fig. 6.**
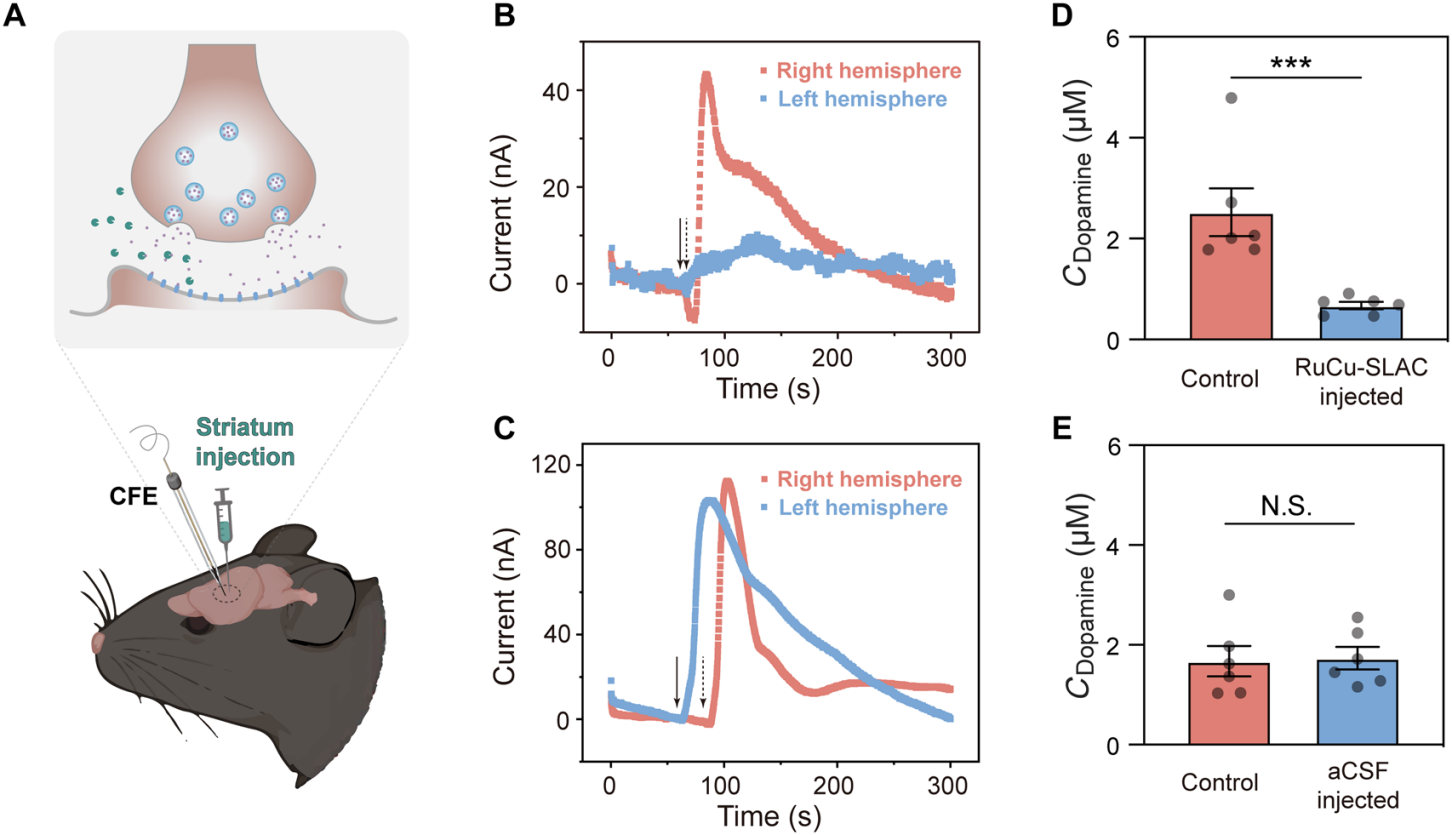
In vivo erasure of evoked dopamine signals. (**A**) Schematic illustration of extrasynaptic regulation of dopamine release from excited dopaminergic neurons by RuCu-SLAC directly injected in the striatum region of the mouse brain. Dopamine currents were simultaneously recorded at the same depths and brain regions of both left and right cerebral hemispheres for parallel comparisons. CFE, carbon fiber electrode. (**B** and **C**) Real-time monitoring of stimulated dopamine release currents in RuCu-SLAC–injected (B) or aCSF-injected (C) mouse brains. Arrows indicate the microinjection of KCl stimulant. (**D** and **E**) Extracellular dopamine concentration changes after stimulated release in RuCu-SLAC–injected (D) or aCSF-injected (E) mouse brains. Each data point represents the dopamine signal recorded on a single mouse. N.S., no significant difference. ***P < 0.001.

One challenge going forward with RuCu-SLAC is to be able to function in living brains. Intriguingly, RuCu-SLAC exerted high catalytic activity and selectivity toward catecholamine oxidation in artificial cerebrospinal fluid (aCSF) mimicking the brain interstitial fluids (fig. S31). Encouraged by the result, we applied RuCu-SLAC for extracellular regulation of secreted dopamine in mouse brains. The striatum region was chosen for in vivo validation because it receives direct projection of dopaminergic neurons in the substantia nigra region. As illustrated in Fig. 6A, an aCSF solution of RuCu-SLAC (10 mg ml^−1^, 3 μl) was administered into the striatum region of the left cerebral hemisphere by microinjection and allowed to diffuse for 1 hour. Afterward, we implanted a carbon fiber microelectrode into the same region and real-time monitored potassium-evoked dopamine transients by in vivo FSCV (detailed in Materials and Methods and fig. S32). Parallelized striatum dopamine signals in the right cerebral hemisphere with no enzyme administration were also recorded as the control.

Representative two-dimensional plots and temporal changing profiles of FSCV data in Fig. 6 (B and C) and fig. S33 present extracellular dopamine dynamics in the striatum. It can be clearly seen that the peak current of striatum dopamine electrooxidation, recorded in the right cerebral hemisphere, sharply increased upon high-potassium stimulation, indicating dopamine burst into the extracellular space. In the striatum of the left cerebral hemisphere after RuCu-SLAC administration, the potassium-evoked dopamine transients were substantially reduced. The average evoked dopamine elevation in the RuCu-SLAC–administered striatum, calculated from FSCV signals recorded at different depths, was 0.5 μM, down below 30% of the control level (2.2 μM) (Fig. 6D). By contrast, aCSF administration without RuCu-SLAC imposes no influence on dopamine signals (Fig. 6E). The measured alteration of interstitial dopamine patterns mirrors the change of synaptic dopamine peak intensity and decay kinetics during neurotransmission, which is a solid demonstration of RuCu-SLAC as a reliable biocatalytic tool for in vivo neuromodulation.

## DISCUSSION

Because of the universal Irving-Williams restriction on protein selection of transition metal cations (Cu^2+^ > Ni^2+^ > Co^2+^ > Fe^2+^ > Mn^2+^) (*31*), it is difficult to construct heteronuclear M-Cu sites (where M refers to noncopper metals) simply by metal replacement. One natural example is given by the heme-copper oxidases that use a bound porphyrin to accommodate an iron center near a copper center. Additional difficulty stems from little knowledge of metal behaviors within the interactive, ever-changing protein ligand fields. Pioneeringly, Lu and colleagues (*19*, *34*, *35*) depicted pictures of building Fe-Fe, Cu-Cu, Fe-Cu, Fe-Mn, and Fe-Zn BNCs in engineered myoglobin scaffolds with remolded binding sites. Recent updates from Choi and Tezcan (*26*) have called attention on multimeric protein interfaces for counter–Irving-Williams selection of transition metals. Here, we propose that ordered installation of coordinately distinct metal cations (e.g., atomic size, binding strength, and equilibrium bond length) within conformation-adaptive interfaces, rather than strictly constraining metal coordination in predesigned steady geometry, can overcome the obstacles in protein design. Because of dynamic and large occupation of the Ru center, only one more Cu is allowed settling in the Ru-coordinated interfacial cleft and shapes the binuclear motif. Compared to the Cu TNC in native laccases, the Ru-Cu BNC can selectively catalyze the O_2_-to-H_2_O conversion at significantly higher TOF values in a pH-universal manner. The transcendental catalytic performance is closely bound up with an interface-confined nonequilibrium structure. By computational and structural investigations, we unravel a self-adaptive mechanism that exploits proximal residue protonation/deprotonation to direct a hybrid pathway for close-to-neutral oxidative catalysis, calling for ultradynamic Ru-Cu coupling to selective strengthening of side-on peroxyl intermediate binding to accelerate reductive O─O cleavage under kinetically retarded conditions.

With this artificial metalloenzyme, we have successfully implemented a nonnatural ROS-free oxidative dopamine catabolism pathway that effectively modulates dopamine neurotransmission at multiple levels of the brain. For whole-cell catalytic modulation, RuCu-SLAC realized sustained down-regulation of both intracellular dopamine synthesis and vesicular release during exocytosis, validating its ability to interrupt presynaptic dopamine packaging and secretion. For in vivo extracellular modulation, RuCu-SLAC displayed rapid and substantial suppression of evoked dopamine transients in mouse brains, underscoring its potential for intervention with dopamine signal transmission to postsynaptic neurons. Compared to traditional pharmacological agents, this enzyme-driven approach offers distinct advantages, including physiological compatibility, reversible and bioregulable modulation, and avoidance of side effects with receptor desensitization.

In summary, this work elucidates an extensible avenue to remolding laccase. In the interface-templated multistep metal ion assembly, the first-step metal binding is universal for elements across the periodic table, which can be stabilized by the resulting dimeric interface to avoid being replaced by Cu^2+^. For example, sequential incubation of *apo*-SLAC with Co^2+^ (inferior to Cu^2+^ in the Irving-Williams series) and Cu^2+^ can make CoCu-SLAC (fig. S34). Besides RuCu-SLAC and CoCu-SLAC, we also made IrCu-SLAC that may feature an Ir-Cu BNC in place of Cu TNC (fig. S35 and table S2). CoCu-SLAC completely loses oxidase activity (fig. S36), while IrCu-SLAC attains the enhancement of oxidase activity at a TOF of 0.20 s^−1^ at pH 7.0 (fig. S37). Although comprehensive investigations of these enzymes are demanded, we are confident that our counter–Irving-Williams strategy has a generalizable applicability to heteronuclear metalloenzymes with refinable active sites and designable functions.

Despite advantages, RuCu-SLAC–based neuromodulation faces translational challenges for clinical applications. First, RuCu-SLAC may impose off-target modulation of epinephrine and norepinephrine signaling. Herein, cellular experiments used undifferentiated PC-12 cells—a well-established model with predominant dopamine synthesis, lower norepinephrine levels, and absence of epinephrine (*36*), ensuring that the recorded alterations of intracellular single-vesicle and exocytotic signals are primarily dopamine-specific. Furthermore, in vivo validation of RuCu-SLAC within dopaminergic brain regions physiologically restricted off-target effects. Second, physiological regulators of RuCu-SLAC performance are complex. For example, endogenous reducing substances like cysteine could postpone dopamine oxidation at the T1 site of RuCu-SLAC (fig. S15). The competitive effects would manifest for biocatalysis inside cells, where cysteine levels are typically among tens to hundreds of micromolar ranges (*37*). Enzyme kinetics of locally injected RuCu-SLAC in the brain shall be less affected, given that the extracellular concentrations of cysteine and other reductants are lowered by several orders of magnitude (*38*). Third, potential cytotoxicity or neurotoxicity could stem from the biocatalysis and biodegradation of RuCu-SLAC. The quinone products of dopamine oxidation can undergo self-cyclization and form a neurotoxic aminochrome in the presence of intracellular ROS. However, glutathione transferases widely expressed in mammals efficiently prevent this metabolic pathway by catalyzing the conjugation of dopa-*o*-quinone and glutathione to form the ROS-resistant 5-*S*-glutathionyldopa, which serves a neuroprotective antioxidant function in neuronal cells (*39*). According to the cell recovery experiments (fig. S28), PC-12 cells can restore the intracellular dopamine synthesis and metabolism at 48 hours following enzyme delivery, implying unimpaired cell functions. Furthermore, cell viability assays show that neither liposomal carriers nor RuCu-SLAC at doses for delivery and neuromodulation would induce cell apoptosis (fig. S38). These results collectively rule out the neurotoxic effects of RuCu-SLAC. Last, as a nontransgenic and biomacromlecular tool, RuCu-SLAC lacks the cell type–specific neuron-targeting and blood-brain barrier (BBB)–penetrating abilities. Protein engineering and formulation are necessitated for its in vivo delivery into the brain or targeted neural tissues.

Future efforts should focus on two directions for translating RuCu-SLAC into clinical neuromodulation therapies. Advanced BBB-penetrating and neuron-targeting delivery strategies are critical (*40*). In light of the precisely designable scaffolds, RuCu-SLAC can be fused with ligands targeting BBB receptors (e.g., transferrin receptor) to enable receptor-mediated transcytosis into the brain and dopamine transporter–binding peptides (e.g., aspartate decapeptide) to target dopaminergic neurons. To minimize peripheral exposure that could degrade administrated proteins or cause off-target side effects, field-responsive liposomal or polymeric nanoparticles functionalized with BBB-shuttling motifs and anti–dopamine transporter antibodies can be exploited to encapsulate RuCu-SLAC and allow spatiotemporally controlled enzyme release in targeted brain regions with the assistance of magnetic or ultrasound stimulation. Stepping further, artificial intelligence–assisted and data-driven rational enzyme redesign is anticipated to improve its in vivo brainwide performance, such as dopamine specificity among catecholamine neurotransmitters, anti-interference ability, structural stability, protease resistance, and prolonged lifetime for treating dopamine-related psychiatric disorders.

## MATERIALS AND METHODS

### Protein template preparation

A protein expression plasmid (pET20b_SLAC) encodes a fusion protein of *apo*-SLAC with a glutathione *S*-transferase (GST) tag and a prescission protease (PPase)–cleavable linker (LEVLFQGP) on the N terminus, which was subjected to codon optimization for the *Escherichia coli* expression system and synthesized by GenScript (Nanjing, China). The expression plasmid encoding recombinant GST-tagged PPase was a gift from M. Wang’s laboratory (Institute of Chemistry, Chinese Academy of Sciences). Expression and preparation of recombinant GST-tagged *apo*-SLAC followed our previously reported method (*28*) with slight modifications. Briefly, BL21(DE3) *E. coli* was transformed with *pET20b_SLAC* and grown on a Luria-Bertani (LB) broth agar plate containing ampicillin (100 μg ml^−1^) at 37°C. A monoclonal colony was inoculated into 150 ml of LB broth medium containing ampicillin (100 μg ml^−1^) and cultured overnight at 37°C (200 rpm). The cell culture was then inoculated into fresh ampicillin-supplemented (100 μg ml^−1^) LB broth medium (1.5 liters × 8) at a volume ratio of 1:100 and grown at 37°C (200 rpm). When OD_600_ (optical density at 600 nm) reached 0.6 to 0.8, isopropyl-β-d-thiogalactopyranoside was added to a final concentration of 0.4 mM to induce protein expression. Cells were further cultured at 25°C (200 rpm) for 18 hours and then harvested by centrifugation at 4000 rpm for 10 min. Cell pellets were resuspended in 50 mM tris-HCl buffer (pH 8.0) containing 150 mM NaCl and the protease inhibitor cocktail at a ratio of 1:10 (w/v) and disrupted by ultrasonication. Insoluble components in the cell lysate were removed by centrifugation at 10,000*g* for 30 min (4°C). The clarified supernatant was loaded onto a preequilibrated a Glutathione Sepharose 4B column by gravity, followed by a thorough wash with 50 mM tris-HCl buffer (150 mM NaCl, pH 8.0). Then, recombinant GST-tagged PPase, prepared as previously reported (*28*), was added onto the column with the bottom exit capped. The mixture was stirred to ensure full contact of the protease and target proteins and allowed to settle down. After overnight in-column incubation at 4°C, untagged *apo*-SLAC was eluted out with the wash buffer and collected in flow pass.

### Artificial metalloenzyme construction

To prepare Ru-SLAC, *apo*-SLAC (2 mg ml^−1^) dissolved in 50 mM tris-HCl buffer (150 mM NaCl, pH 8.0) was incubated with 1 mM RuCl_3_‧3H_2_O (Macklin) at 4°C overnight under gentle shaking. Precipitates were removed by centrifugation at 10,000*g* for 30 min. The colored supernatant was diluted with tris-HCl buffer to bring the NaCl concentration down to 25 mM and then loaded onto a DEAE-Sepharose FF column at a flow rate of 3 ml min^−1^ for anion-exchange chromatographic purification by the AKTA chromatography system (Cytiva). Ru-SLAC fractions were collected with a NaCl gradient (50 to 500 mM) in 50 mM tris-HCl buffer (pH 8.0) and combined for concentrating in an Amicon centrifugal filter (30-kDa cutoff) tube (Merck Millipore). The concentrated sample was last subjected to gel filtration through a Superdex-75 10/300 GL size exclusion column with 50 mM tris-HCl buffer (150 mM NaCl, pH 8.0) at a flow rate of 0.5 ml min^−1^. The protein purity of each collected fraction (1 ml) was verified by SDS–polyacrylamide gel electrophoresis. Fractions solely displaying the Ru-SLAC band were combined, concentrated, and stored in 100 μl of aliquots at −80°C for further characterizations. To prepare the heteronuclear RuCu-SLAC, Ru-SLAC (4 mg ml^−1^) in 50 mM tris-HCl buffer (150 mM NaCl, pH 8.0) was separated into 1 ml in an Eppendorf tube and incubated with 1 mM CuCl_2_ under gentle shaking for 2 hours (37°C). Implementation of Cu^2+^ into Ru-SLAC was assessed by UV-vis absorbance from 350 to 700 nm on a microplate reader (BioTek Synergy H1M) or a UV-vis spectrometer (Thermo Evolution 220). The resulting mixture was collected and diluted with tris-HCl buffer to decrease the concentration of Cu^2+^, followed by concentrating in an Amicon centrifugal filter (30-kDa cutoff) tube (Merck Millipore). The concentrated sample was last subjected to gel filtration by the same procedure as Ru-SLAC purification. Collected fractions of RuCu-SLAC were verified by SDS–polyacrylamide gel electrophoresis and stored in 100 μl of aliquots at −80°C after concentrating.

### Crystallography and structure refinement

Crystallization of RuCu-SLAC was conducted at 16°C by the sitting drop method in wells containing 100 mM bis-tris (pH 6.5), 2% (v/v) polyethylene glycol monomethyl ether 550, and 1.8 M ammonium sulfate. The drops made with a well solution contained RuCu-SLAC (20 mg ml^−1^). The successfully grown crystals were collected and flash frozen in liquid nitrogen for subsequent structural determination. X-ray diffraction data were collected at 100 K and 2.10 Å with a complementary metal-oxide semiconductor detector on the BL19U beam line of the Shanghai Synchrotron Research Facility. The protein structure was built by molecular replacement with native SLAC [Protein Data Bank (PDB) ID: 3CG8] as the starting model. Structural refinement was performed in Phenix (*41*), and model rebuilding was done in Coot (*42*). Crystallography and refinement statistics are listed in table S4.

### XAFS characterization

The RuCu-SLAC sample for XAFS characterization was prepared by exchanging the buffer to 2.5 mM tris-HCl buffer (pH 8.0) by size exclusion chromatography on a PD-10 desalting column (Cytiva, US), followed by freeze-drying to powder form. XAFS characterization was conducted at the BL11B beamlines at the Shanghai Synchrotron Radiation Facility. Cu K-edge EXAFS and EXANES (extended XANES) spectra of the RuCu-SLAC sample and standards were collected under transmission mode, while Ru K-edge EXAFS and EXANES spectra were collected under fluorescence mode. Data analysis and fitting were done by the IFEFFIT software package using the Athena and Artemis modules.

### MD simulations

The crystal structure of RuCu-SLAC was used to set starting atomic coordinates for simulating protein dynamics in solution. The Ru-Cu BNC was restrained in a single-bonded mode to avoid its escape during MD simulation by assigning real bonds of Ru-H287 and Cu-H289. T1 Cu was strictly restrained in a tricoordinated geometry by assigning real bonds of Cu-H231, Cu-C288, and Cu-H293. Partial atomic charges and CHARMM force field parameters for bonds, angles, dihedrals/impropers, and nonbonded interactions (table S1) were calculated by Multiwfn (*29*) and Sobtop (*30*). Protein structure files were generated with the psfgen module in VMD (*43*) and parameterized using the CHARMM22 (*44*, *45*) force field for protein backbone and calculated force field parameters for the Ru-Cu BNC and T1 Cu. The RuCu-SLAC dimer was solvated in a TIP3P (*46*) water box with a distance of 15 Å to the box edge and neutralized by adding explicit Na^+^ and Cl^−^ ions (150 mM). MD simulations by NAMD (*47*) was conducted in four steps. First, only water molecules and Na^+^ and Cl^−^ ions were minimized with the protein solute fixed. Second, all atoms were subjected to unconstrained minimization in an NVT ensemble. Third, the system was warmed up to 310 K through gradient heating at an increment of 1 K every 200 fs and equilibrized in an NPT ensemble under periodic boundary conditions at a constant pressure of 1 atm and temperature of 310 K for 200 ps. Last, production MD was run for 10 ns. The time step was 2 fs, and atom trajectories were recorded every 2 ps. Distance measurements, occupancy calculation, and structure cluster analysis were accomplished in UCSF Chimera (*48*).

### DFT calculations

For O_2_ BE calculations, O_2_-bound complexes were built from MD-identified possible motif conformations of Ru(II) or Cu(I) centers and subjected to unrestricted open-shell geometry optimization and frequency calculation with Gaussian 16 (*49*) by the PBE0 functional (*50*) in combination of D3BJ dispersion correction (*51*). The Lanl2TZ(f) ECP basis set (*52*) was adopted for Ru and Cu atoms, and the 6-311G** basis set was adopted for all nonmetal atoms. All complexes were calculated with various multiplicities to find the lowest-energy geometry. Metal-centered motifs without O_2_ and free O_2_ molecules were also separately calculated by the same method. The O_2_ BE can be obtained by the following equation

$$\text{BE}=E_{\text{oxy}-\text{complex}}-E_{\text{complex}}-E_{\text{dioxygen}}$$

where *E* refers to the electronic energy. For cluster-model DFT calculations, the **PI**_**Ru**_ cluster was built by extracting the most dominant Ru-centered motif **4** and proximal histidine residues (H102/158/234) from the MD-simulated **FO** ensemble, replacing one water ligand by O_2_ and resigning Ru(III) to Ru(II). The **PI**_**Ru-Cu**_ cluster was built by extracting the most dominant oxyRu-N_H104_-Cu motif with eight directly coordinated histidine residues and one proximal tyrosine (Y108). Starting from these two clusters, possible transition states and intermediates were explicitly considered to deduce the most reasonable reaction pathway. All clusters were optimized by fixing metal atoms and non-H atoms of residues, leaving H atoms of residues as well as all atoms of water and O_2_ unfrozen. The same PBE0 functional and basis set combination as the BE calculation was applied for cluster calculation. The implicit solvent model of PCM was used to mimic the dielectric environment of the protein interfacial cleft. To help with self-consistent field convergence, additional Gaussian key words were used: opt = (cartesian,gdiis,recalc = 5), scf = (novaracc,noincfock), and int = acc2e = 12. Molecular orbitals were inspected and visualized by Multiwfn (*29*).

### Homogeneous characterization of oxidase activity

Dopamine oxidation was first assessed by UV-vis absorbance scanning in 50 mM sodium phosphate buffer containing 1 mM dopamine and RuCu-SLAC (0.02 mg ml^−1^) or native SLAC (0.02 mg ml^−1^) for 5 min. To assay catalytic kinetics, the reaction was monitored with 1 mM dopamine and RuCu-SLAC (0.05 mg ml^−1^) or native SLAC (0.05 mg ml^−1^) at 480 nm with a molar extinction coefficient of 3058 M^−1^ cm^−1^ for quinone products (*53*). ABTS assays were conducted in 50 mM sodium phosphate buffer containing 1 mM ABTS and RuCu-SLAC (0.05 mg ml^−1^) or native SLAC (0.05 mg ml^−1^). Production of ABTS^•+^ was monitored at 420 nm with a molar extinction coefficient of 34,000 M^−1^ cm^−1^ (*54*). Buffers of different pH levels were used to assay pH-dependent oxidase activity. Amounts of RuCu-SLAC or native SLAC added to assay cocktails were quantified by T1 Cu characteristic absorbance at 590 nm with a molar extinction coefficient of 4400 M^−1^ cm^−1^ (*18*). By this means, active RuCu-SLAC can be unified with native SLAC for precise activity comparison, and those T1 Cu–deficient inactive enzymes were excluded.

### Electrochemical characterization of O_2_-reducing activity

Before electrochemical characterization, RuCu-SLAC was modified onto the electrode by our reported method to achieve direct electron transfer between the T1 Cu of RuCu-SLAC and the electrode (*23*, *55*). Briefly, single-walled CNTs (SWNTs) were dispersed in ethanol, coated onto the electrode surface, and dried under a lamp. Then, 20 μl of tris-HCl solution containing RuCu-SLAC (20 mg ml^−1^) and 20% ethanol was drop-cast onto the SWNT-modified electrode and air dried. An additional Nafion coating was applied to avoid the fall-off of the enzymatic layer during rotation. For hydrodynamic measurements, a RuCu-SLAC/SWNT–modified E6R2 rotating ring-disk electrode (glassy carbon disk OD = 5.61 mm, Pt ring OD = 7.92 mm; PINE Instruments, US) was used as a working electrode controlled by WaveDriver 200 Integrated Bipotentiostat (PINE Instruments, US). A Ag/AgCl electrode was used as a reference electrode, while Pt wire was used as a counter electrode. Linear sweep voltammograms were collected at various rotating speeds from 50 to 1200 rpm with a scan rate of 5 mV s^−1^ in 50 mM O_2_-saturated tris-HCl (150 mM NaCl, pH 6.0). The Pt ring electrode was biased at +0.50 V versus Ag/AgCl to record the oxidation current of H_2_O_2_ generated from the disk electrode. The electron transfer number (*n*) was calculated by the following equation

$$n=4\times \frac{I_{d}}{I_{d}+\frac{I_{r}}{N}}$$

where *I*_d_ is the disk current, *I*_r_ is the ring current, and *N* is the current collection efficiency of the Pt ring determined to be 0.37.

### Operando spectroelectrochemical characterization

In situ Raman spectroscopic experiments were performed with the mIRage system (Photothermal Spectroscopy Corporation), all conducted in a custom thin-layer electrochemical microcell (Beijing Scistar), with a CNT-modified glassy carbon electrode serving as the working electrode and Ag/AgCl (3 M KCl solution) and platinum wires serving as the reference electrode and counter electrode, respectively. The glassy carbon electrode was polished with Al_2_O_3_ powder and then sequentially sonicated in ethanol and Milli-Q (18.2 megohms·cm) water. Next, CNT dispersion (in ethanol) was dropped onto the surface of the glassy carbon electrode repeatedly to ensure that it covered the entire surface. Tris-HCl buffer (50 mM tris, 150 mM NaCl, pH 8.0) with or without protein [RuCu-SLAC (4.5 mg ml^−1^) or *apo*-SLAC (4.5 mg ml^−1^)] was used as an electrolyte solution. For each measurement, a voltage was applied for at least 5 min to ensure that the surface of the electrode reached a stable state. The excitation wavelength of the laser was 532 nm. The probe power and resolution were 72% and 1200, respectively. The integral time was 30 s.

### Protein formulation for cellular delivery

RuCu-SLAC was labeled with FITC to visualize protein delivery. Briefly, 6 mg of RuCu-SLAC dissolved in 950 μl of sodium bicarbonate solution (0.1 M, pH 9.0) was mixed with 50 μl of freshly prepared FITC solution (20 mg ml^−1^ in dimethyl sulfoxide). Then, the reaction mixture was stirred at room temperature for 1 hour with protection from light. The FITC-labeled RuCu-SLAC was purified on a PD-10 desalting column (Cytiva, US). To formulate FITC-labeled RuCu-SLAC for cellular delivery, LD NPs (1 mg ml^−1^ in 25 mM sodium acetate buffer, pH 5.2) and FITC-labeled RuCu-SLAC (1 mg ml^−1^ in 2.5 mM tris-HCl, pH 8.0) were mixed at a mass ratio of 1:3 in 10 mM acetate buffer (pH 5.2) and homogenized for 15 min to obtain LD-SLAC NPs.

### Cell culture

PC-12 cells were purchased from the National Infrastructure of Cell Line Resource (Beijing, China) and maintained in RPMI 1640 (Gibco) medium supplemented with 10% horse serum (Procell), 5% fetal bovine serum, and 1% penicillin/streptomycin in the presence of 5% CO_2_. Before experiments, PC-12 cells were subcultured and seeded in poly-l-lysine–precoated 48-well plates or coverslips for at least 4 hours before use.

### Cellular delivery of RuCu-SLAC

PC-12 cells were treated with LD-SLAC NPs at a final proteinic concentration of 20 μg ml^−1^. After 12-hour incubation at 37°C, the cells were washed thoroughly with Dulbecco’s phosphate-buffered saline (PBS) and harvested for flow cytometry analysis on a Beckman Coulter CytoFLEX (Beckman Coulter, US). To investigate the cell distribution of LD-SLAC NPs, PC-12 cells were seeded in glass-bottom cell culture dishes (NEST Biotechnology, Wuxi, China) for at least 4 hours, followed by treatment with LD-SLAC NPs for 12 hours at 37°C at a final proteinic concentration of 12 μg ml^−1^. At the end of incubation, PC-12 cells were washed with Dulbecco’s PBS and imaged by confocal laser scanning microscopy on an OLYMPUS FV1000-IX81.

### Vesicle probe fabrication

Carbon fiber electrodes for single-vesicle measurement were fabricated by established methods (*56*). Briefly, 10-μm carbon fibers (Dagan Co., Minneapolis, US) were inserted into a glass capillary (1.5-mm outer diameter, 0.84-mm inner diameter) and then pulled into two separately by a P-2000 micropipette puller (Sutter Instrument Co., Novato, US). The exposed end of carbon fiber was cut to the tip of the micropipette, and the fiber-glass interface was sealed with epoxy. The electrodes were transferred to a drying oven and cured at 60°C overnight and then polished with a microgrinder (EG-400, Narishige, Japan) into disk electrodes. As for nanotip carbon fiber electrode fabrication, the exposed end of carbon fiber was first etched by ethanol flame before epoxy sealing. All electrodes (disk and nanotip) were subjected to electrochemical activation 12 hours before experiment following the procedure: amperometry at +1.5 V (versus Ag/AgCl) for 80 s and then cyclic voltammetry from 0 to 1.0 V at a scan rate of 50 mV s^−1^ in 1 M NaOH, controlled by a potentiostat (CHI 1030C, CH Instrument Inc., Shanghai, China). The electrochemical reactivity of electrodes was tested by cyclic voltammetry in PBS buffer containing a 100 μM dopamine solution at pH 7.4. Electrodes showing dopamine responses and stable currents were used for investigation.

### Single-vesicle electrochemistry

Coverslips carrying PC-12 cells were rinsed three times with standard physiological saline (135 mM NaCl, 5 mM KCl, 1 mM MgCl_2_, 2 mM CaCl_2_, 10 mM glucose, and 10 mM Hepes) and then placed under an inverted microscope (TS100, Nikon, Japan) in a Faraday cage for electrochemical recording. The carbon fiber electrode was held at +780 mV (versus Ag/AgCl) by a patch-clamp amplifier (700B, Axon Instrument, CA). The signal output was filtered at 300 Hz using a low-pass Bessel filter and digitized at 10 kHz (1550A, Axon Instrument, CA, US). Single-vesicle electrochemical recordings were performed as reported previously (*57*). For on-cell measurements, the disk microelectrode was used as a working electrode and positioned on the cell membrane surface for microamperometry recording. A micropuffer system (RCP-2B, Inbio Inc., Wuhan, China) was introduced for the 5-s injection of 70 mM KCl solution (78 mM NaCl, 70 mM KCl, 2 mM CaCl_2_, 1 mM MgCl_2_, 10 mM glucose, and 10 mM Hepes) to stimulate cell exocytosis. For intracellular experiments, the working electrode was changed to the nanotip microelectrode, which was inserted into cells carefully with a micromanipulator (MP-225, Sutter Instrument Co., Novato, US). All current transients recorded in on-cell and intracellular measurements respectively represent the burst of catecholamine molecules released during a single exocytotic event and the total content of catecholamine in each vesicle, which are oxidized into catecholamine-quinone at the surface of the carbon fiber electrode. For data processing and analysis, single-vesicle currents were first treated through a filter of 600 Hz (binomial sm.) and then analyzed by Igor software. Five times the standard deviation of the noise was set as the threshold to identify the peaks, and only the traces with more than 10 validated peaks were reserved. The number of dopamine molecules (*N*_molecules_) stored in a single vesicle or released in a single exocytotic event was quantified by Faraday’s equation

$$N_{\text{molecules}}=\frac{Q}{\mathrm{nF}}$$

where *Q* is the charge from the time integral of vesicle exocytotic spikes or vesicle impact spikes, *n* is the number of electrons exchanged in the oxidation reaction (2 for catecholamines), and *F* is Faraday’s constant (96,485 C mol^−1^). The mean of the median calculated from single cells was used. By this way, we can minimize the impact of cell-to-cell variations, as the value is less sensitive to outliers. All the data are represented as the means ± SEM. The *P* value was calculated using GraphPad Prism 9. Pairs of datasets were compared with the two-tailed Mann-Whitney rank-sum test.

### In vivo validation

Animal surgeries and experiments were approved and supervised by the Animal Care and Use Committee at Beijing Normal University. Male C57BL/6J (6 to 8 weeks) mice purchased from Health Science Center, Peking University, were housed on a 12:12-hour light-dark schedule with food and water ad libitum. Before in vivo tests, carbon fiber microelectrodes (7 μm in diameter) for in vivo FSCV were fabricated as previously described (*58*). Briefly, the exposed end of a carbon fiber sealed in a glass micropipette was cut to 100 to 150 μm by a surgery scalpel under a microscope. They were subjected to electrochemical activation by applying +1.5 V (versus Ag/AgCl) for 20 s in 1 M NaOH, followed by repeated cyclic voltammetry in an aCSF solution (126 mM NaCl, 2.4 mM KCl, 0.5 mM KH_2_PO_4_, 0.85 mM MgCl_2_, 27.5 mM NaHCO_3_, 0.5 mM Na_2_SO_4_, and 1.1 mM CaCl_2_, pH 7.4) until a stable voltammogram was obtained. For validation of RuCu-SLAC function in vivo, animals were positioned onto a stereotaxic frame after being anesthetized with isoflurane (4% induction and 2% maintenance) through a R520 gas pump (RWD, China). A carbon fiber microelectrode and a polypropylene tube was coimplanted into the striatum region (anteroposterior, 0 mm; lateral, 3 mm from bregma; ventral, 4 mm from dura). A RuCu-SLAC local injection was performed by exogenous microinfusion through a silica capillary tube at a rate of 1.0 μl min^−1^ for 3 min, and the tube was implanted 0.1 mm above the depth of electrodes. FSCV was performed using a commercial instrument (ElProScan ELP-3 model with EPC10 USB triple potentiostat, HEKA Electronik GmbH, Lambrecht/Pfalz, Germany) with prepared carbon fiber microelectrodes as working electrodes. Miniaturized Ag/AgCl wire and Pt wire were embedded in subcutaneous tissue on the brain and used as reference and counter electrodes, respectively. All FSCV recordings were conducted within a potential range from −0.4 to 1.1 V at a scan rate of 400 V s^−1^ and repeated every 200 ms. Between scans, the electrode was held at −0.4 V.
