# Supplementary material for: Remolding laccase for whole-cell and in vivo modulation of dopamine signal
Source: Sci Adv. 2025 Oct 22;11(43):eady3842. doi: 10.1126/sciadv.ady3842 (PMC12542965; doi:10.1126/sciadv.ady3842)
Supplement: Supplementary file 1 — Figs. S1 to S38 Tables S1 to S5 [file sciadv.ady3842_sm.pdf]

Science Advances
AAAS

Supplementary Materials for

Remolding laccase for whole-cell and in vivo modulation of dopamine signal

Xiaoti Yang *et al.*

Corresponding author: Fei Wu, wufei317@bnu.edu.cn; Ping Yu, yuping@iccas.ac.cn;
Lanqun Mao, lqmao@bnu.edu.cn

Sci. Adv. **11**, eady3842 (2025)
DOI: 10.1126/sciadv.ady3842

This PDF file includes:

Figs. S1 to S38
Tables S1 to S5

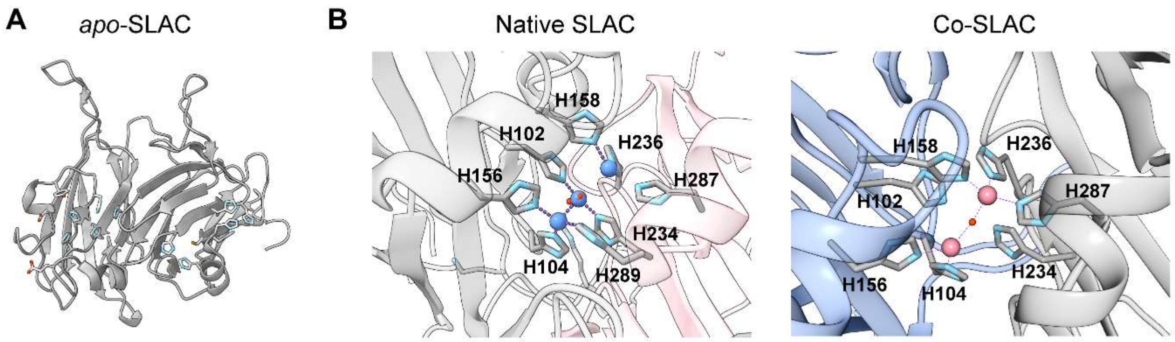

Fig. S1. Structures of SLAC-scaffolded enzymes. (A) Crystal structure of apo-SLAC monomer (PDB ID: 3CG8) with surface hisditine residues highlighted. (B) Structures of the T2/T3 Cu trinuclear cluster formed at native SLAC dimer interface (PDB ID: 3CG8) and an oxygen-bridged dicobalt cluster formed at Co-SLAC dimer interface (PDB ID: 7W6V).

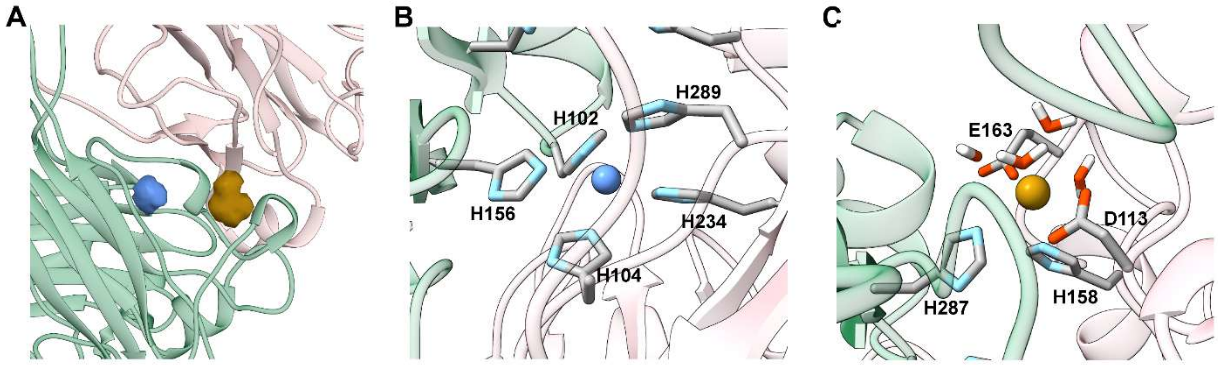

Fig. S2. Interactions of SLAC template and transition metal cations. (A) Simulated atomic occupancy of Cu^{2+} and Ru^{3+} at SLAC dimer interface in non-bonded mode. (B,C) Simulated interactions of Cu^{2+} (B) and Ru^{3+} (C) with interfacial residues in non-bonded mode.

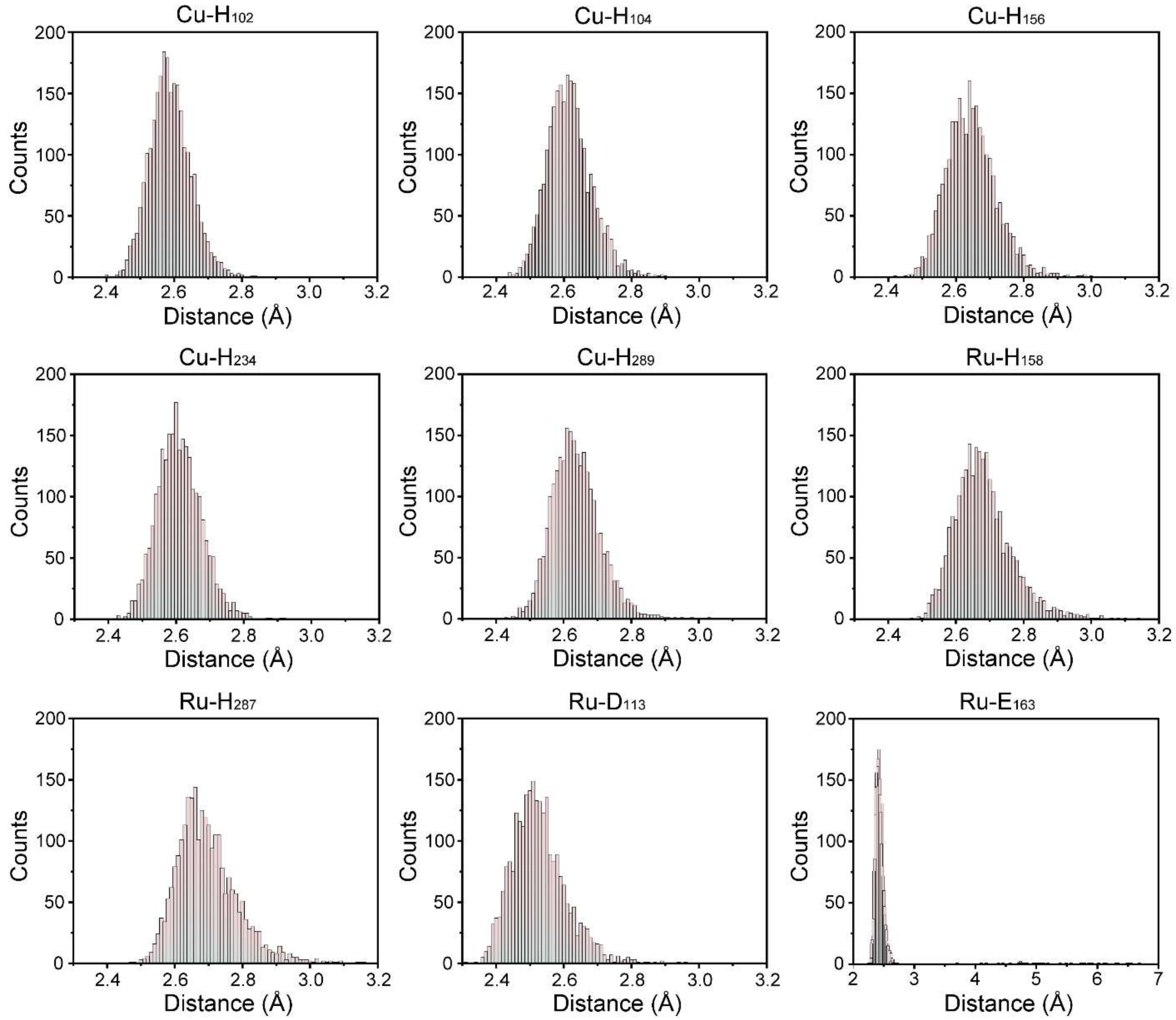

Fig. S3. Simulated metal-residue distances. Distributions of distances between Cu^{2+}/Ru^{3+} and proximal coordinable residues were measured from non-bonded MD trajectories.

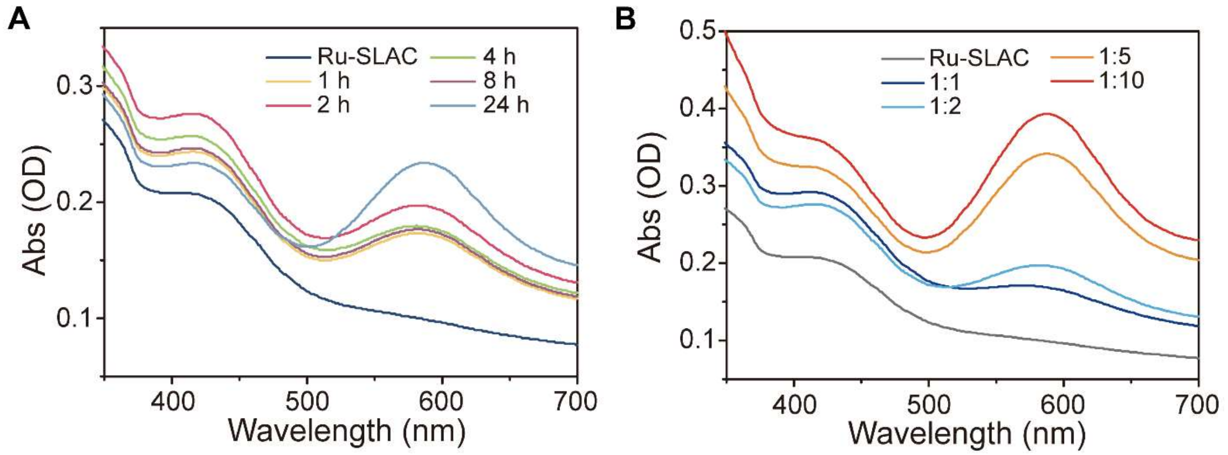

Fig. S4. UV-vis absorbance spectra of Ru-SLAC dimers with Cu^{2+}. (A) 1 mM $CuCl_2$ at different incubation time lengths. (B) Incubation for 2 h with $CuCl_2$ at different Ru:Cu stoichiometric ratios. Buffer, 50 mM tris-HCl buffer (150 mM NaCl, pH=8.0).

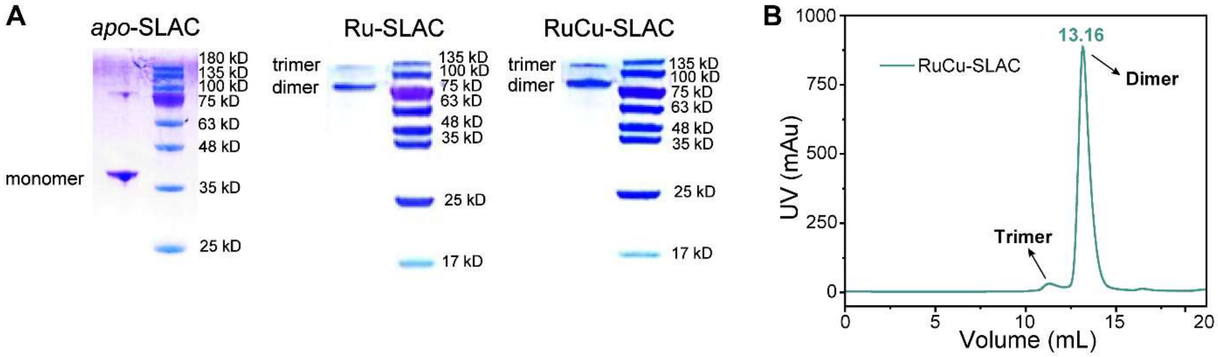

Fig. S5. Purity and oligomerization degree of RuCu-SLAC. (A) Gel electrophoresis. (B) gel filtration.

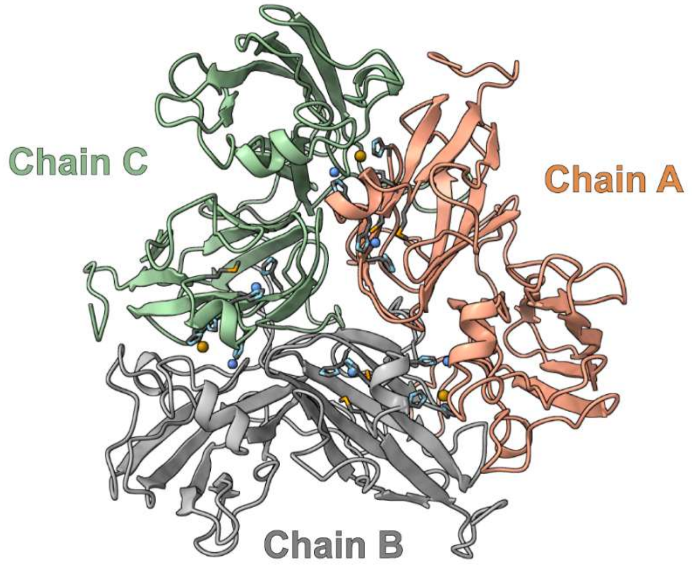

Fig. S6. Trimeric form of RuCu-SLAC. Three monomers were head-to-tail assembled by the interfacial Ru-Cu BNC.

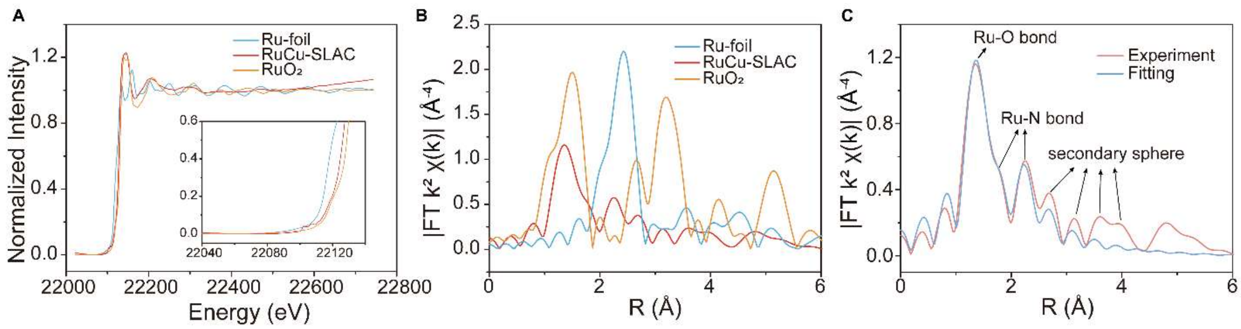

Fig. S7. Fine-structure determination of Ru center in RuCu-SLAC. (A) Ru K-edge XANES spectra. (B) Fourier-transformed (FT) k^2-weighted EXAFS spectra. (C) Comparison of experimental and calculated FT k^2-weighted EXAFS spectra of the Ru center in R space.

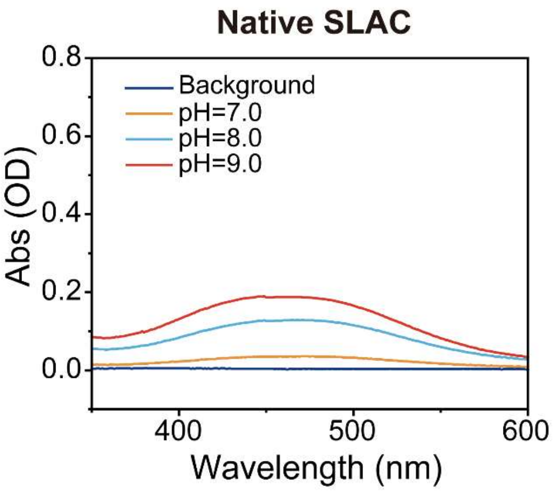

Fig. S8. Enzyme activity toward dopamine. Absorbance changes (5 min) of *o*-quinone product at 480 nm in assay cocktails of 1.35 μM native SLAC or RuCu-SLAC, 1 mM dopamine, 50 mM sodium phosphate and 150 mM NaCl at pH 7.0, 8.0 and 9.0.

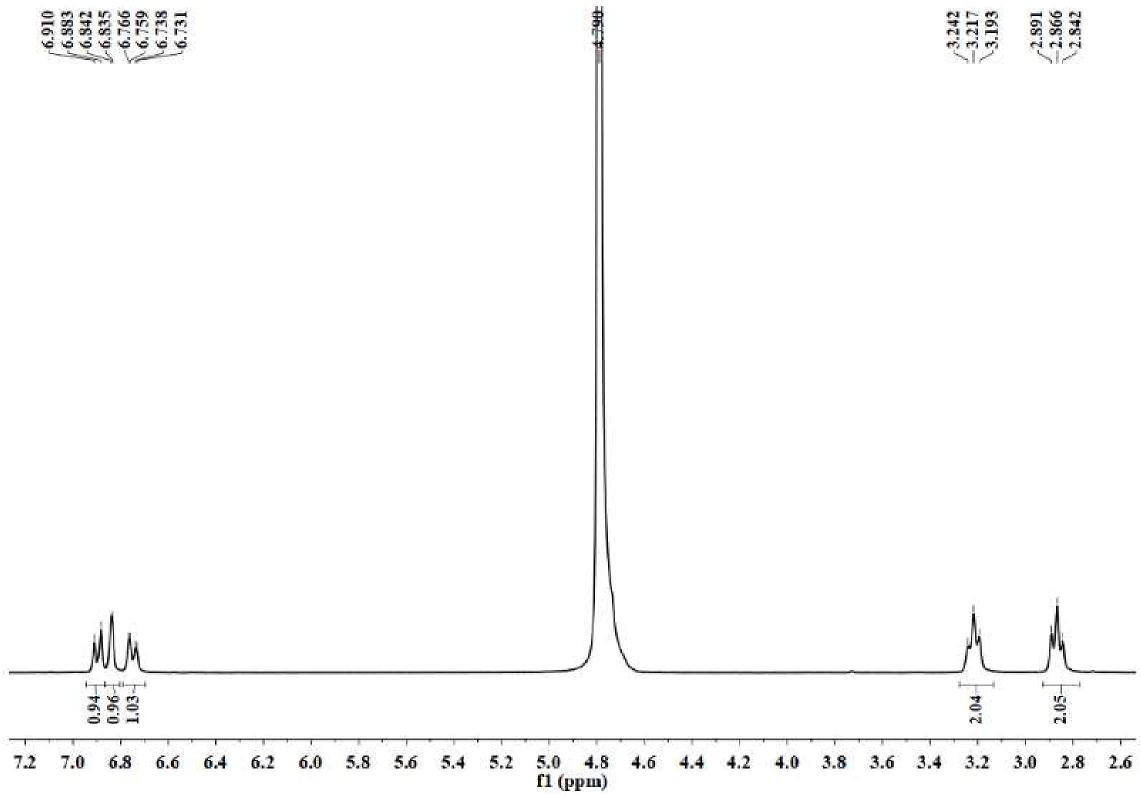

Fig. S9. Characterization of enzymatic products. ^{1}H nuclear magnetic resonance (NMR) spectroscopy of *o*-quinone produced by a mixture of 0.01 mg mL^{-1} RuCu-SLAC and dopamine in D_2O.

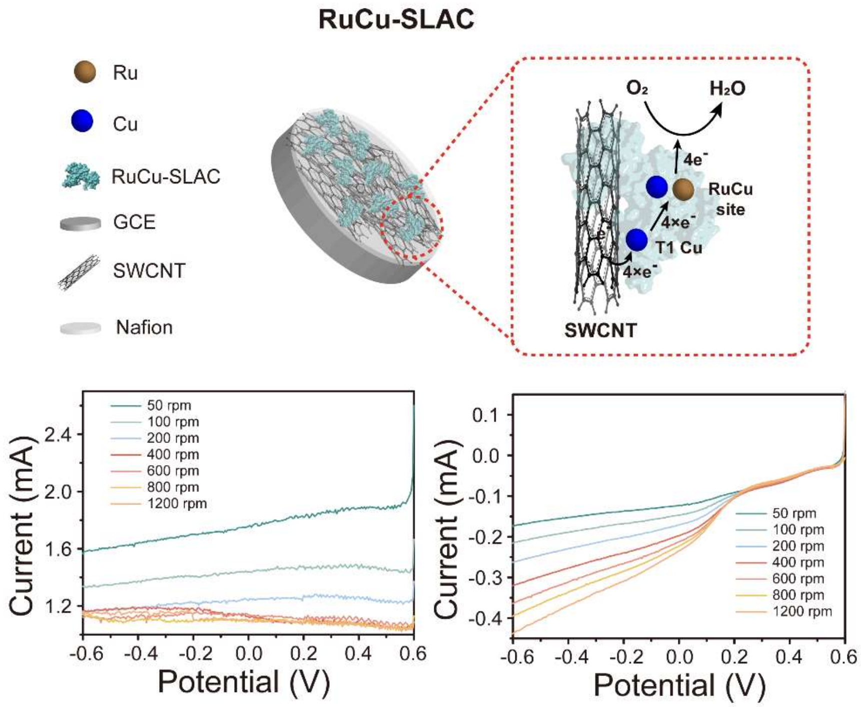

Fig. S10. Rotating ring-disk electrode (RRDE) characterization of RuCu-SLAC-catalyzed ORR. RuCu-SLAC was immobilized on a single-walled carbon nanotube (SWCNT)-modified glassy carbon disk electrode, circled by a Pt ring electrode for detecting H_2O_2 delivered from two-electron ORR at the disk electrode. Left, Linear sweep voltammograms of Pt ring electrode collected at varying rotating speeds. Right, Linear sweep voltammograms of RuCu-SLAC-functionalized disk electrode collected at varying rotating speeds.

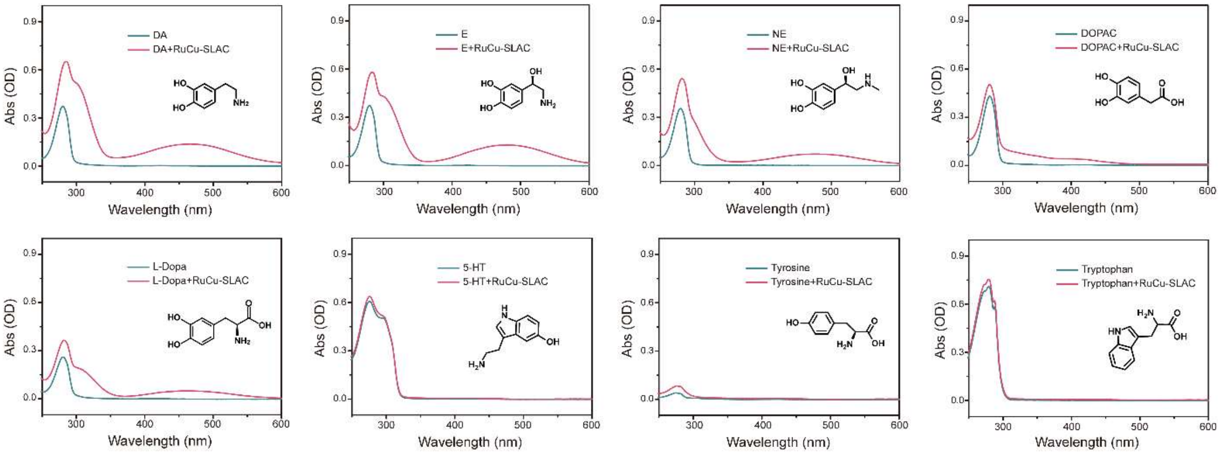

Fig. S11. Substrate scope. Biocatalytic activities of RuCu-SLAC towards monoamines and related metabolites.

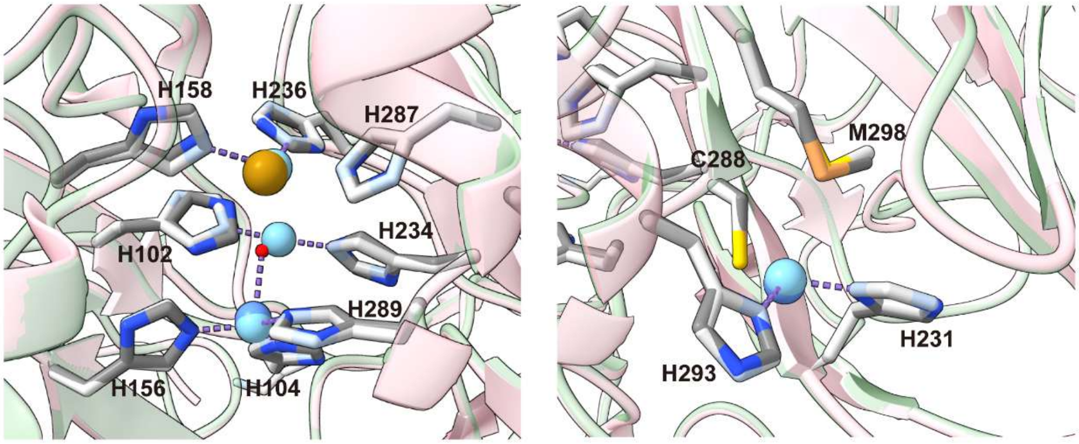

Fig. S12. Structural comparison of RuCu-SLAC with native SLAC. Crystal structures of RuCu-SLAC and native SLAC (PDB ID: 3CG8) were aligned at RMSD < 0.5 Å. Left, Superimposed structures of Ru-Cu BNC and T2/T3 Cu TNC. Right, Superimposed structures of two T1 Cu sites. Cu atoms of RuCu-SLAC and native SLAC are respectively colored in dark blue and light blue. T1 Cu sites show high structural consistency in both enzymes.

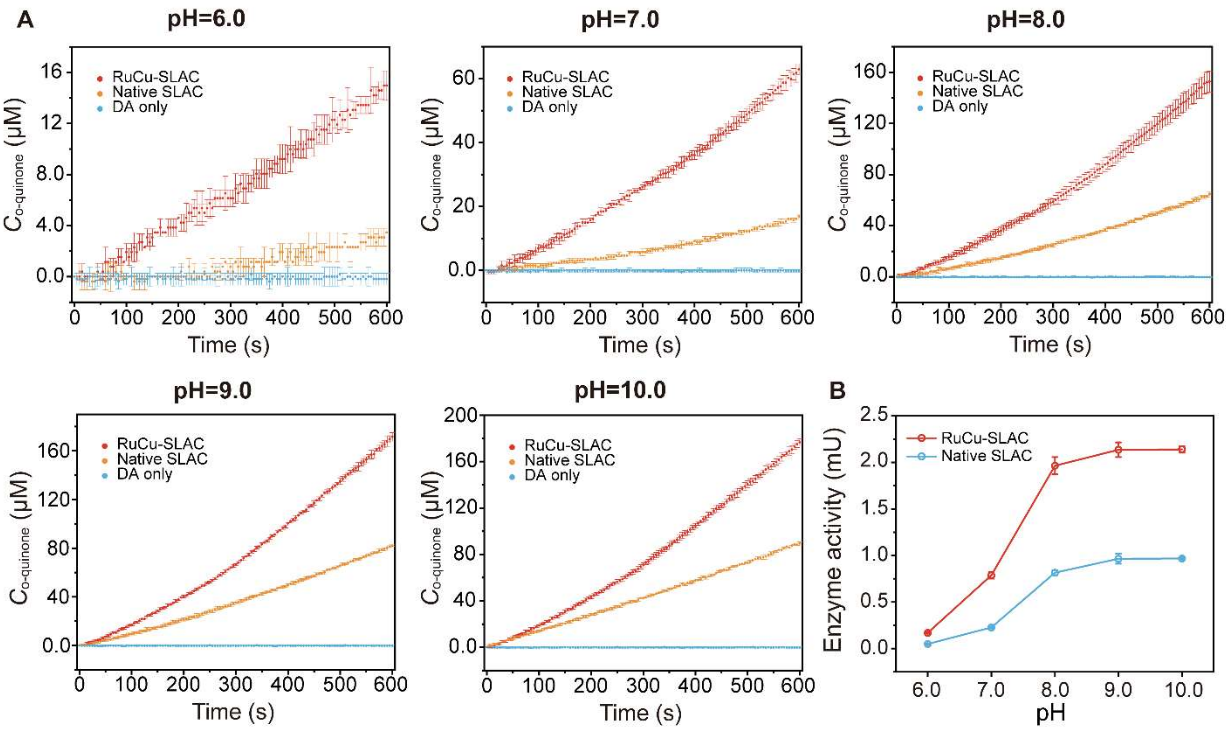

Fig. S13. Dopamine oxidation assays of RuCu-SLAC and native SLAC. (A) Temporal profiles of *o*-quinone production with RuCu-SLAC, native SLAC or enzyme-free dopamine solutions at pH 6.0-10.0. DA, dopamine. (B) Catalytic activities of RuCu-SLAC and native SLAC towards dopamine oxidation at varying pHs.

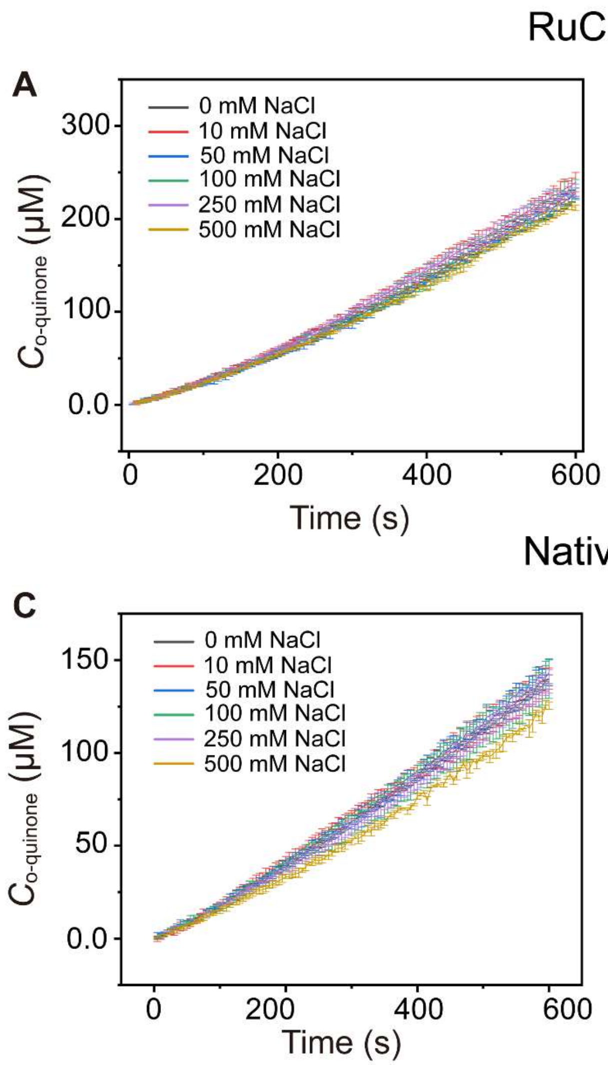

Fig. S14. Chloride inhibition of RuCu-SLAC and native SLAC. (A,C) Temporal profiles of *o*-quinone production with 0.1 mg/mL RuCu-SLAC (A) or native SLAC (C) in phosphate buffer containing different concentrations of NaCl at pH 8.0. (B,D) Normalized activities of RuCu-SLAC (B) or Native SLAC (D) towards dopamine oxidation in the presence of NaCl at varying concentrations.

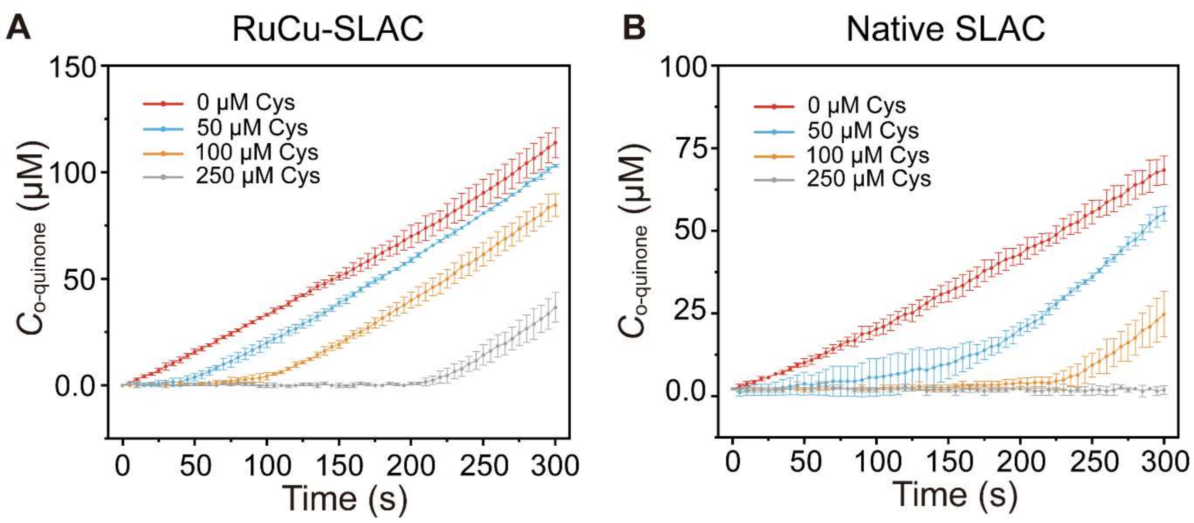

Fig. S15. Cysteine inhibition of RuCu-SLAC and native SLAC. (A,B) Temporal profiles of *o*-quinone production with 0.1 mg/mL RuCu-SLAC (A) or native SLAC (B) in PBS containing different concentrations of cysteine at pH 8.0. Cys, L-cysteine.

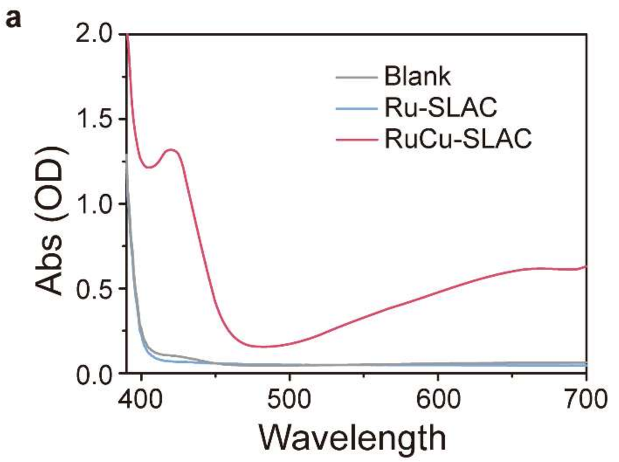

Fig. S16. ABTS oxidation assays of RuCu-SLAC and native SLAC. (A) The UV-vis absorbance spectra of ABTS with RuCu-SLAC or Ru-SLAC. (B) Catalytic activities of RuCu-SLAC and native SLAC towards ABTS oxidation at varying pHs.

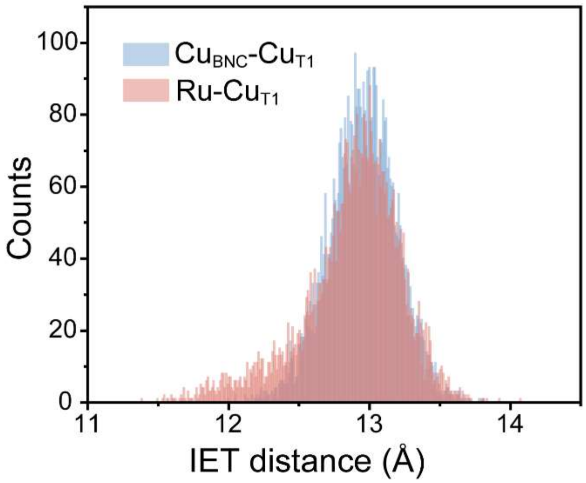

Fig. S17. Distributions of IET distances in solvated RuCu-SLAC. Distances of RuCu-BNC and T1 Cu were extracted from a 10 ns MD trajectory (single-bonded mode), measured every 2 ps.

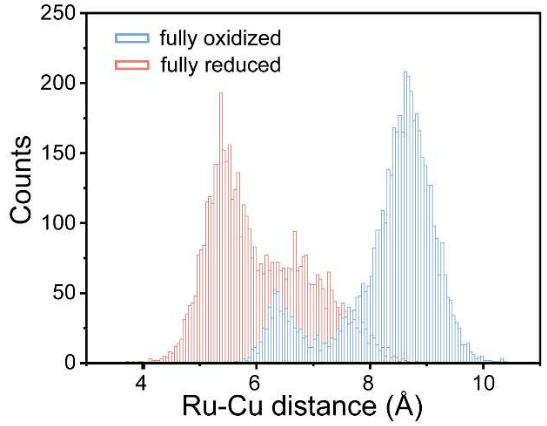

Fig. S18. Simulated Ru-Cu distances. Distribution histograms of Ru^{III}-Cu^{II} (fully oxidized) and Ru^{II}-Cu^{I} (fully reduced) distances at dimeric interface

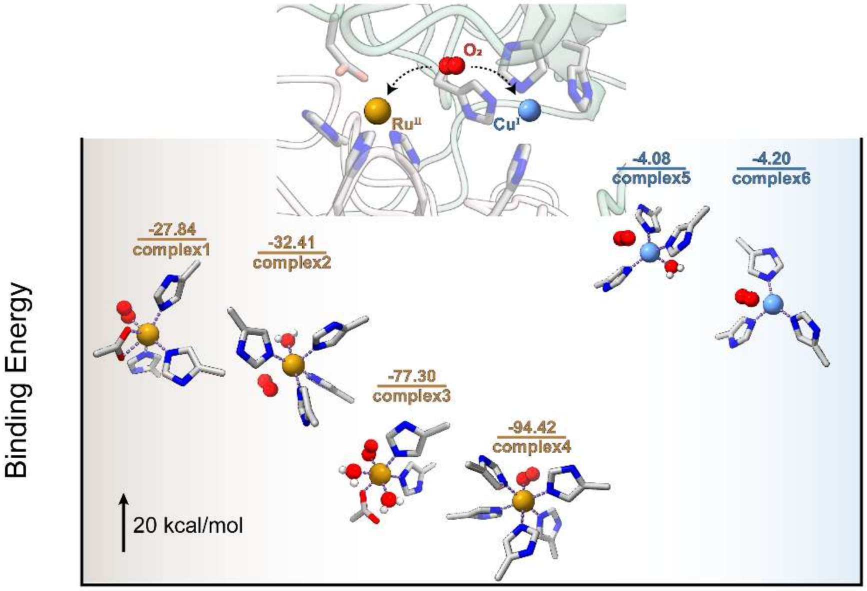

Fig. S19. DFT-calculated electronic energies for O_2 binding at non-coupled Ru^{II}-Cu^{I} BNC. The oxyRu^{II} and oxyCu^{I} centers were modeled by simplified metal-ligand complexes built on possible motif structures suggested by MD simulations.

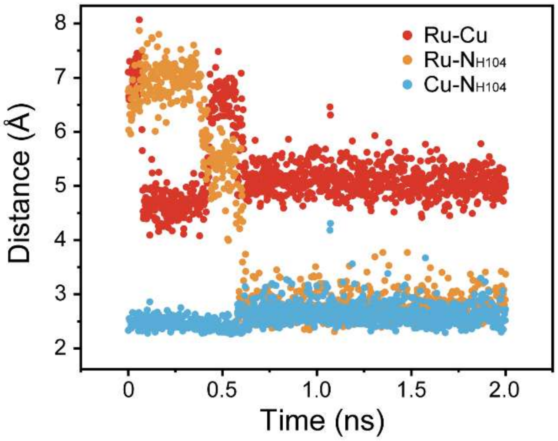

Fig. S20. Time-dependent distance changes of fully reduced Ru^{II}-Cu^{I}.

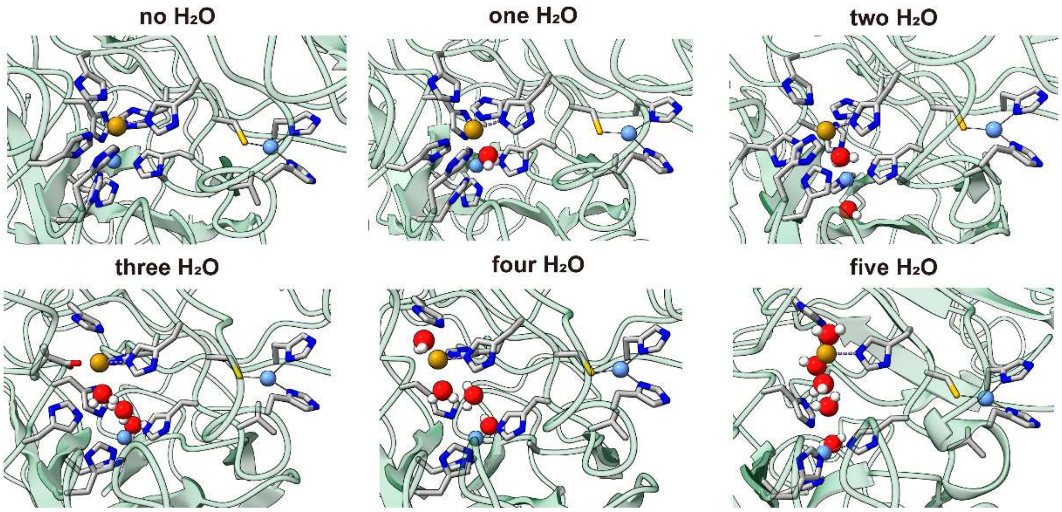

Fig. S21. Simulated water dynamics at RuCu-SLAC interface. MD snapshots of Ru-Cu BNC with water molecules entering into the interfacial cleft and aligning linearly between metal centers through possible H-bonds.

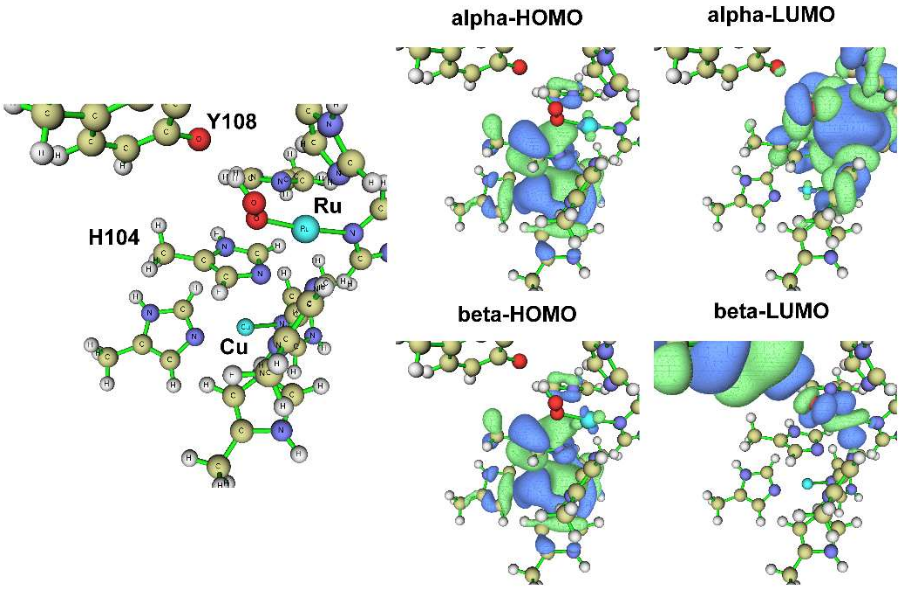

Fig. S22. DFT-computed molecular orbitals of [RuCu]-OOH intermediate. Left, Clear structure of H104-bridged Ru-Cu cluster with side-on Ru-OOH oriented towards Y108. Unrestricted open-shell optimization was performed to deduce [RuCu]-OOH intermediate from **PI$_{\mathbf{Ru\text{-}Cu}}$**. Right, Computed HOMOs and LUMOs for alpha and beta electrons in [RuCu]-OOH intermediate.

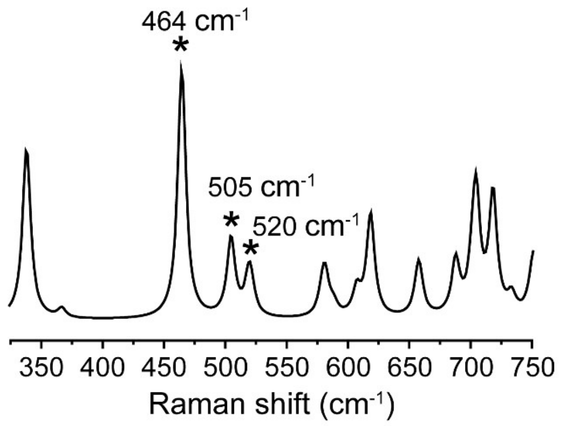

Fig. S23. DFT-Calculated Raman spectrum of the Ru-Cu BNC at the resting FO state. Peaks of Ru–O stretching and out-of-plane bending of Ru-bound water ligand in the motif **4** configuration are marked by asterisks.

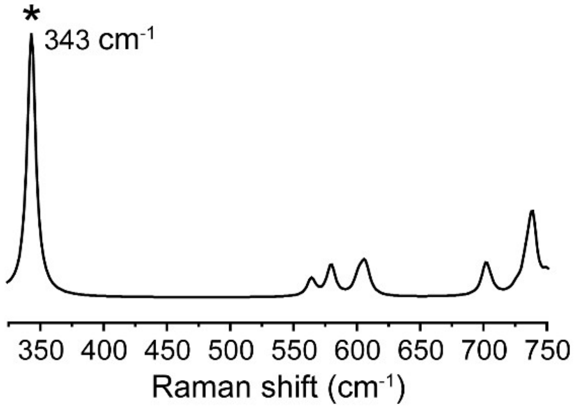

Fig. S24. DFT-Calculated Raman spectrum of the proposed PI$_{Ru\text{-}Cu}$ intermediate. Peak of Ru–O stretching is marked by asterisk.

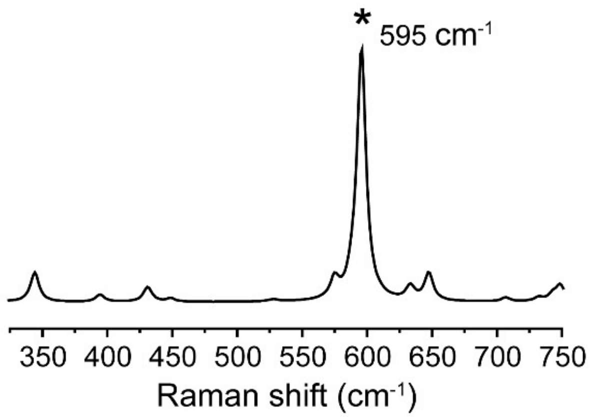

Fig. S25. DFT-Calculated Raman spectrum of the proposed mononuclear Ru^{II}-OH intermediate. Peak of Ru–O stretching is marked by asterisk.

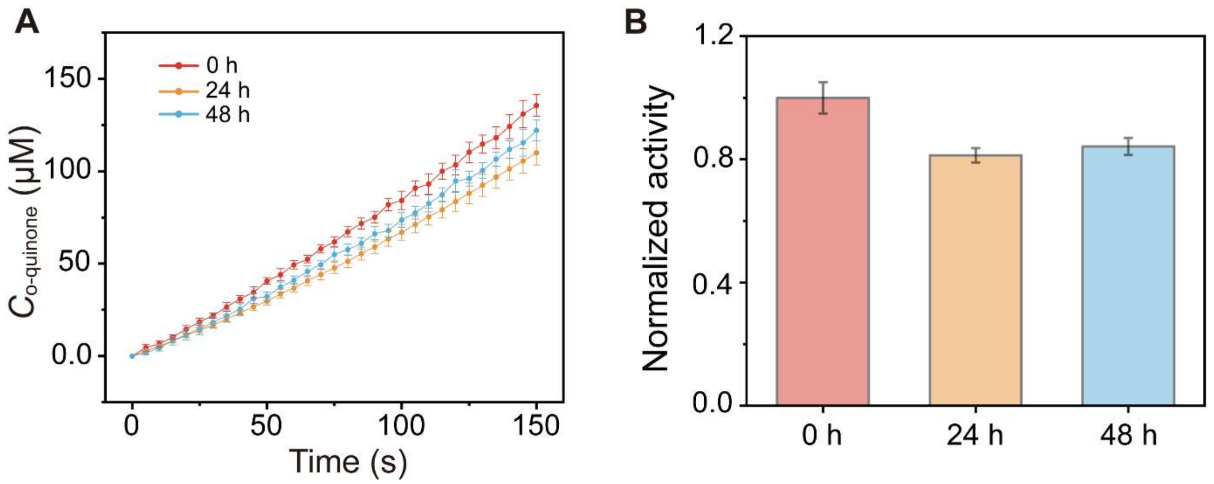

Fig. S26. Stability assessment of RuCu-SLAC. (A) Temporal profiles of *o*-quinone production with 0.1 mg/mL RuCu-SLAC in artificial cerebrospinal fluid (aCSF, pH 7.2~7.4) after incubation for 0 h, 24 h and 48 h at 4℃. Dopamine concentration, 1 mM. (B) Normalized catalytic activities of RuCu-SLAC.

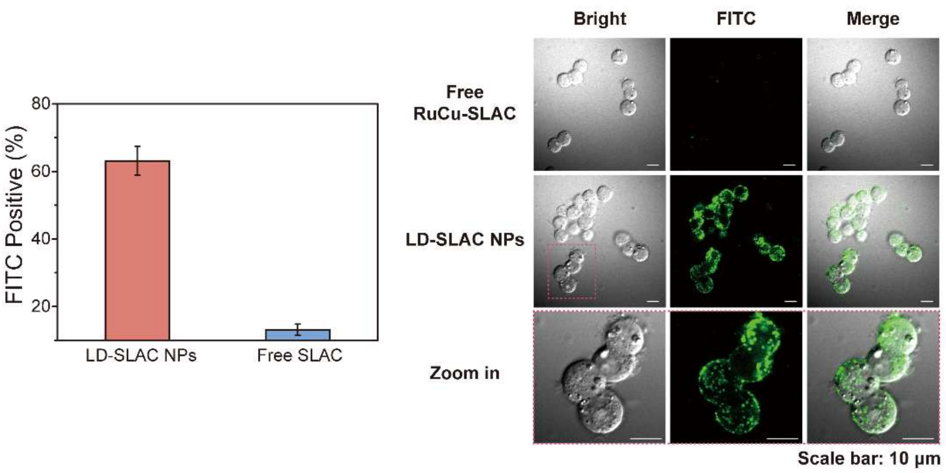

Fig. S27. Intracellular delivery of RuCu-SLAC. Left, Quantitative analysis of RuCu-SLAC delivery efficiency by counting FITC-positive cells. Right, Confocal microscopic imaging of PC-12 cells incubated with FITC-labeled RuCu-SLAC in free form or liposome-encapsulated form.

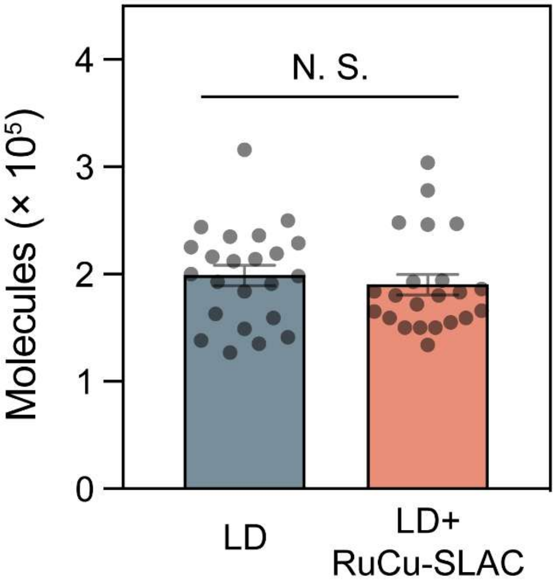

Fig. S28. Recovery of intracellular dopamine. Total numbers of dopamine molecules stored in single cytosolic vesicles were measured before and after RuCu-SLAC delivery for 48 h. Each data point represents the mean value of all vesicles recorded inside a single cell. N. S., no significant difference.

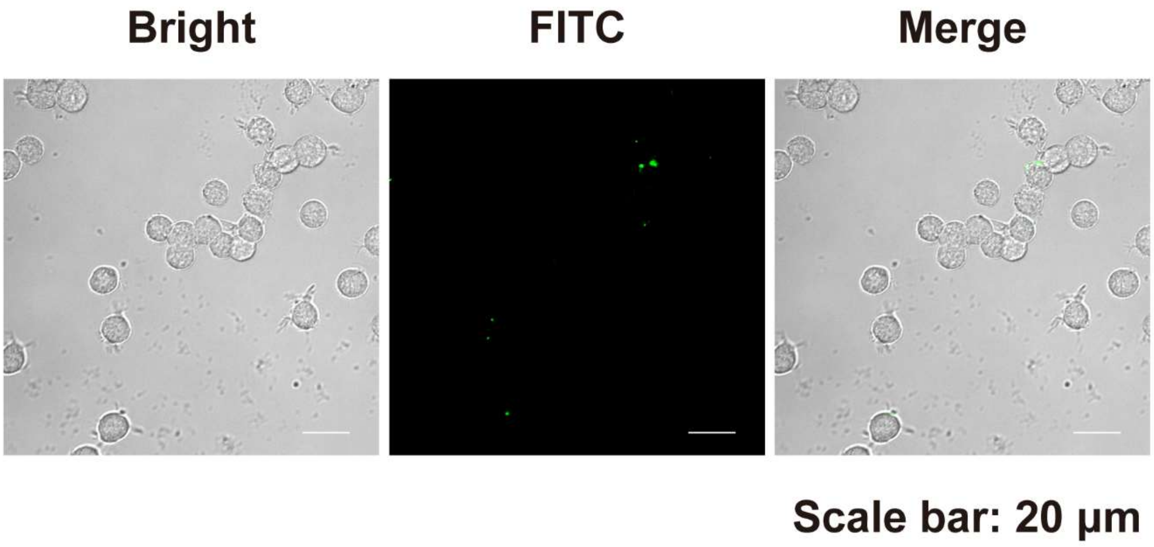

Fig. S29. Cytosolic removal of RuCu-SLAC. Confocal microscopic imaging of PC-12 cells was performed at 48 h after delivery of FITC-labeled RuCu-SLAC.

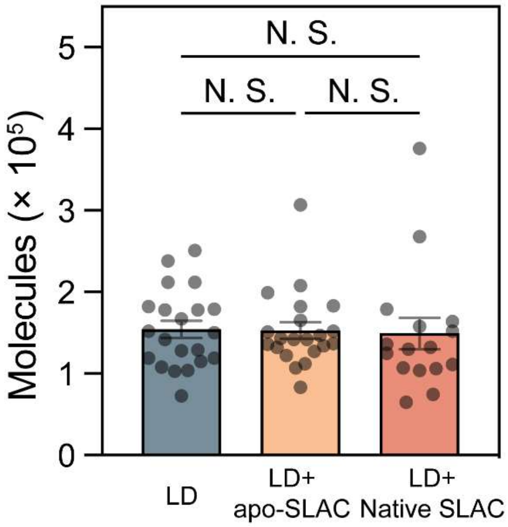

Fig. S30. Intracellular effects of native SLAC. Total numbers of dopamine molecules released in single vesicle exocytosis of PC12 cells were measured after delivery of LD carriers, apo-SLAC or native SLAC for 12 h. Each data point represents the mean value of all exocytotic events recorded on a single cell. N. S., no significant difference.

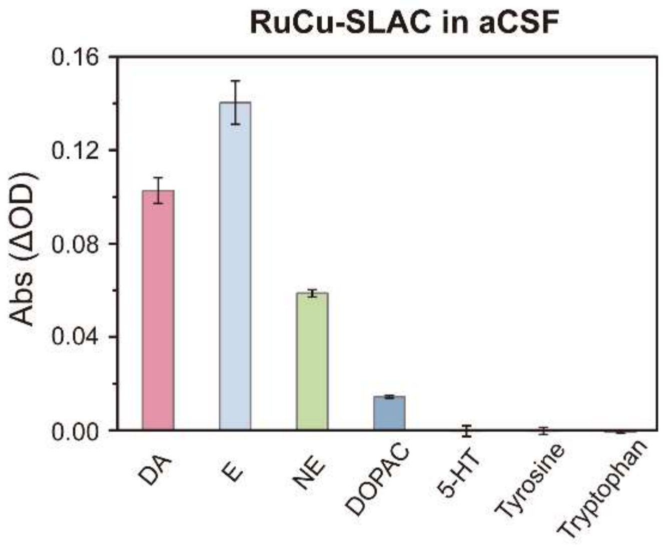

Fig. S31. Selectivity of RuCu-SLAC towards catecholamine neurotransmitters in aCSF. Oxidative catalytic activities of RuCu-SLAC were assayed by steady-state UV-vis absorbance change of quinone products generated from different phenolic biomolecules in the nervous system.

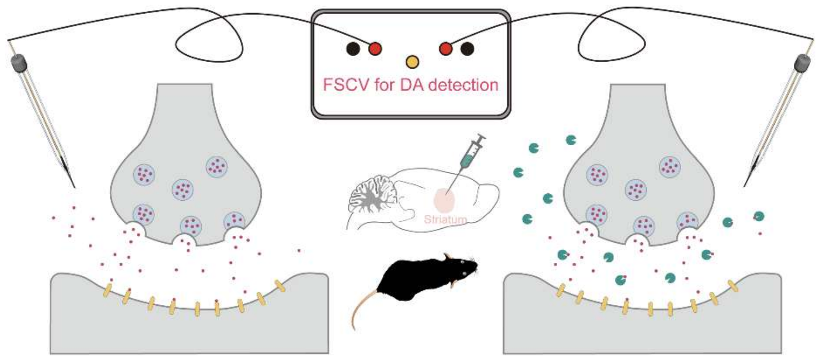

Fig. S32. In vivo dopamine modulation. Schematic illustration shows in vivo fast-scan cyclic voltammetry for striatum dopamine signals detection after local delivery of RuCu-SLAC for extracellular catalysis of dopamine oxidation.

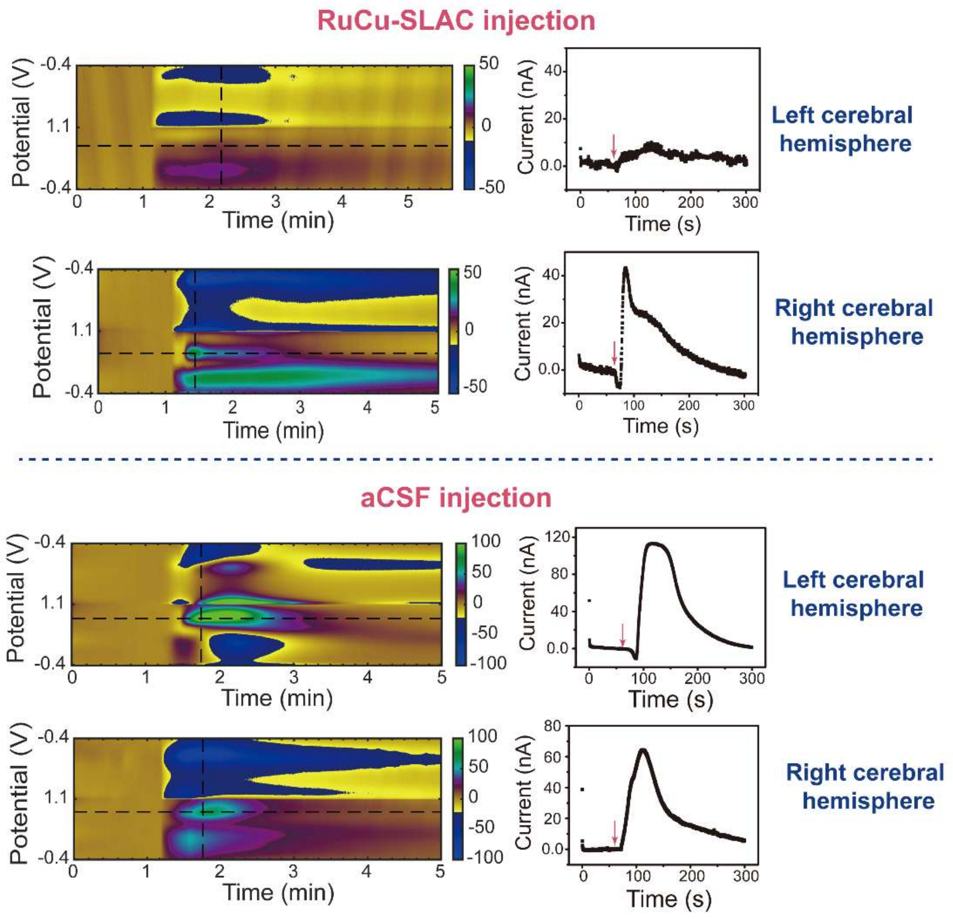

Fig. S33. Electrochemical evaluation of *in vivo* RuCu-SLAC efficacy on dopamine neurotransmission. Left, Representative 2D plots of FSCV data. Crossbars indicate the current peak of dopamine. Right, Representative temporal profiles of current changes recorded at the peak potential, extracted from the crossbar points of 2D FSCV plots. Artificial cerebrospinal fluid (aCSF) containing RuCu-SLAC was directly injected into the striatum region on the left cerebral hemisphere to consume dopamine molecules secreted into the extracellular space or synaptic clefs. A microelectrode was locally implanted to real-time monitor extracellular dopamine concentration change. Another microelectrode was implanted at the same brain region on the righ cerebral hemisphere to record dopamine signals as parallel test. Microinjection of 70 mM KCl triggered excitation of dopaminergic neurons to secrete dopamine, reflected by the sharp current increase. In the presence of RuCu-SLAC, the stimulated current was largely reduced. For control experiments, blank aCSF was injected and stimulated dopamine currents were not affected.

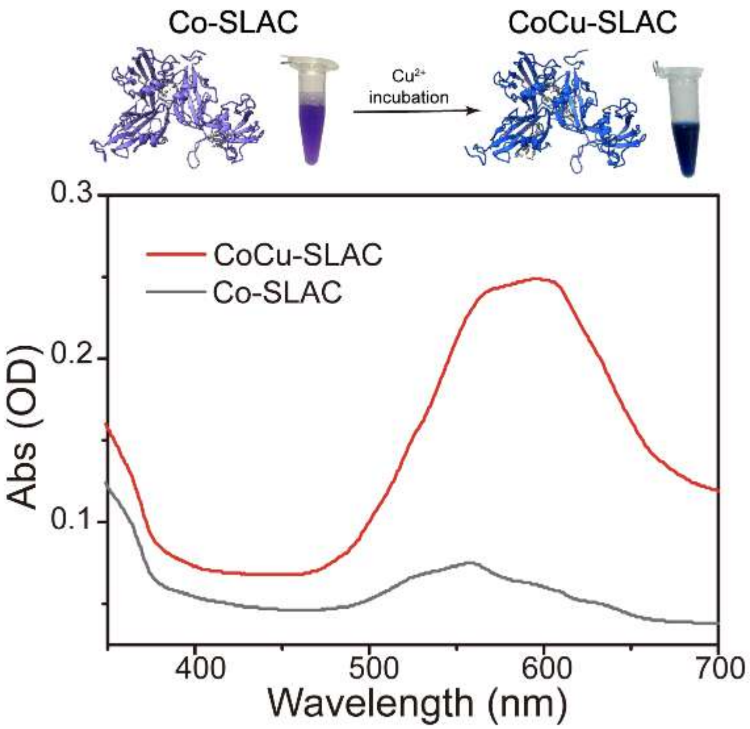

Fig. S34. Characterization of CoCu-SLAC. Schematic illustration shows CoCu-SLAC construction, monitored by UV-vis absorbance spectroscopy.

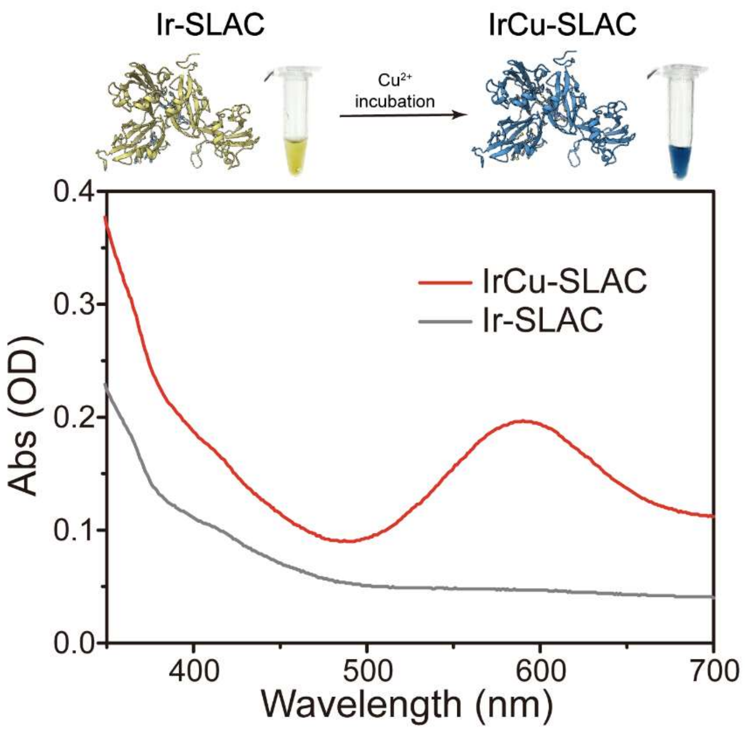

Fig. S35. Characterization of IrCu-SLAC. Schematic illustration shows IrCu-SLAC construction, monitored by UV-vis absorbance spectroscopy.

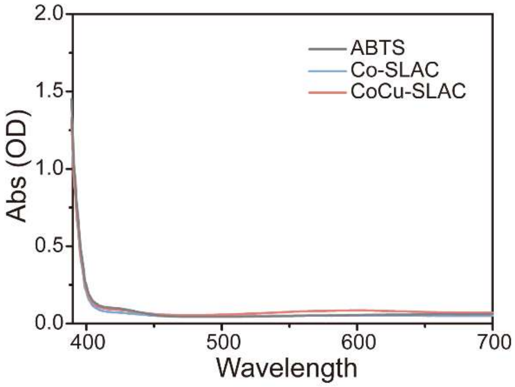

Fig. S36. Activity of CoCu-SLAC toward ABTS. UV-vis absorbance spectra of ABTS-O_2 reaction were collected in assay cocktails of CoCu-SLAC, ABTS, 50 mM sodium phosphate and 150 mM NaCl at pH 7.0. ABTS self-oxidation was also monitored as control.

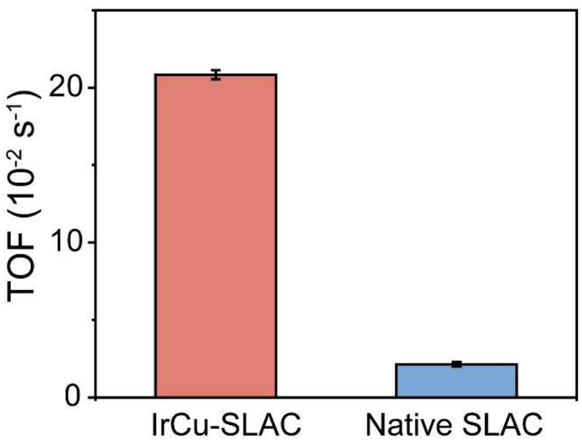

Fig. S37. Enzyme kinetics of IrCu-SLAC. TOF value of IrCu-SLAC is compared with that of native SLAC in catalyzing dopamine oxidation at pH 7.0.

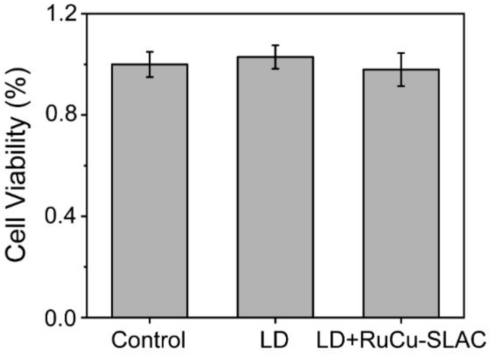

Fig. S38. Cytotoxic evaluation of RuCu-SLAC. Cell viability assays of LD carriers and RuCu-SLAC-encapsulated LD NPs on PC12 cells were preformed at 48 h after delivery.

Table S1. Computed CHARMM force field parameters for $Ru^{III/II}$ and $Cu^{II/I}$ coordination at dimer interface.

Bonds	K_b (kcal·mol^{-1}·Å^{-2})	b_o (Å)
Ru^{III}–NR2	56.28	2.17
Ru^{II}–NR2	74.66	2.12
Ru^{II}-OM	43.75	2.19
Cu^{II}–NR2	63.63	2.02
Cu^{I}–NR2	120.33	1.91
**Angles**	K_θ (kcal·mol^{-1}·rad^{-2})	θ_o (degree)
Ru^{III}–NR2–CPH1	52.95	126.37
Ru^{III}–NR2–CPH2	54.45	125.80
NR2–Ru^{III}–NR2	113.98	178.53
Cu^{II}–NR2–CPH1	23.19	127.23
Cu^{II}–NR2–CPH2	23.30	126.42
NR2–Cu^{II}–NR2	15.03	102.91
Ru^{II}–NR2–CPH1	24.37	128.47
Ru^{III}–NR2–CPH2	25.08	125.01
Cu^{I}–NR2–CPH1	15.91	125.87
Cu^{I}–NR2–CPH2	15.74	127.24
Ru^{II}–OM–OM	13.88	142.71
NR2–Ru^{II}–OM	44.41	173.27
**Dihedrals/Impropers**	K_ξ (kcal·mol^{-1}·rad^{-2})	ξ_o (degree)
CPH1-CPH1-NR2-Ru^{III}	59.01	180.00
NR1-CPH2-NR2-Ru^{III}	52.24	-180.00
HR3-CPH1-NR2-Ru^{III}	23.43	0.00
HR1-CPH2-NR2-Ru^{III}	23.76	0.00
CPH1-NR2-Ru^{III}-NR2	0.11	30.41
CPH2-NR2-Ru^{III}-NR2	0.11	-140.59
NR2-Ru^{III}-NR2-CPH1	0.12	27.21
NR2-Ru^{III}-NR2-CPH2	0.12	-152.80
CPH1-CPH1-NR2-Cu^{II}	20.46	180.00
NR1-CPH2-NR2-Cu^{II}	19.71	-180.00

HR3-CPH1-NR2-Cu^{II}	13.36	0.00
HR1-CPH2-NR2-Cu^{II}	13.83	0.00
CPH1-CPH1-NR2-Ru^{II}	19.30	180.00
NR1-CPH2-NR2-Ru^{II}	19.87	-180.00
HR3-CPH1-NR2-Ru^{II}	12.47	0.00
HR1-CPH2-NR2-Ru^{II}	13.41	0.00
CPH1-CPH1-NR2-Cu^{I}	10.70	180.00
NR1-CPH2-NR2-Cu^{I}	10.23	-180.00
HR3-CPH1-NR2-Cu^{I}	7.85	0.00
HR1-CPH2-NR2-Cu^{I}	7.40	0.00
NR2-Ru^{II}-OM-OM	0.66	-155.16
CPH1-NR2-Ru^{II}-OM	0.52	-58.02
CPH2-NR2-Ru^{II}-OM	0.52	122.79
**Nonbonded**	ε (kcal/mol)	$r_{min/2}$ (Å)
Ru	-0.056	1.48
Cu	-0.005	

Table S2. Determined stoichiometry of MCu-SLAC (M=Ru or Ir) by ICP-MS.

Protein name	**M** content (‰)	**Cu** content (‰)	Ratio of **M**:**Cu**
RuCu-SLAC	2.57	6.23	0.94:3.4
IrCu-SLAC	10.0	5.11	1:1.56

Note 1: The Ru:Cu ratio of RuCu-SLAC is close to 1:3, suggesting that each dimer hosts one Ru atom and three Cu atoms. This is in good agreement with the crystal structure that a Ru-Cu BNC locates at the dimer interface while two mononuclear Cu sites separately locate on each monomeric subunit.

Note 2: The Ir:Cu ratio of IrCu-SLAC is close to 1:1.5. Since the number of Cu atoms should be an integer, the most probable stoichiometry is that each dimer hosts two Ir atoms and three Cu atoms. However, it is unlikely that the interface can accommodate another Ir site in addition to a Ir-Cu BNC. Instead, the other Ir site may be a surface-confined mononuclear site.

Table S3. Enzyme kinetics of RuCu-SLAC and native SLAC towards dopamine oxidation in physiological pH range.

RuCu-SLAC

pH	K_m (mM)	V_{max} (μM·s^{-1})	K_{cat} (min^{-1})
6.0	2.83±0.56	0.73±0.06	16.2±1.3
7.0	1.42±0.24	0.96±0.05	21.3±1.1
8.0	1.20±0.44	1.78±0.23	39.6±5.1

Native SLAC

pH	K_m (mM)	V_{max} (μM·s^{-1})	K_{cat} (min^{-1})
6.0	4.32±0.63	0.23±0.09	5.11±2.00
7.0	3.08±0.43	0.83±0.06	18.4±1.3
8.0	0.95±0.11	0.70±0.03	15.6±0.6

Note 3: All kinetic parameters were determined in air-saturated PBS containing 0.1 mg/mL enzymes.

Table S4. Predicted pKa values of ionizable residues of RuCu-SLAC.

Residue	pK(int)	pK(1/2)
Gly36	7.053	6.993
Arg40	12.403	>12.000
Glu47	4.296	4.04
Arg49	10.823	>12.000
His50	6.293	6.158
Lys52	10.366	11.469
Tyr54	11.02	>12.000
Glu56	4.78	4.36
Lys57	9.673	11.664
Asp60	3.976	3.36
Tyr65	13.377	>12.000
Glu68	4.57	3.673
Lys69	10.036	10.734
Lys71	10.345	11.203
Glu80	5.323	2.779
Glu83	5.273	<0.000
Asp85	3.91	0.34
His88	6.635	6.053
Glu90	4.962	3.525
Asp96	4.399	3.009
Arg98	11.057	>12.000
His102 *	6.46	5.828
His104 *	6.199	4.328
Asp107	4.903	0.918
Tyr108 *	11.121	>12.000
Glu109	4.227	3.32
Asp113 *	3.926	1.139
Lys119	10.317	10.682
Asp121	4.025	0.895
Glu123	4.422	3.943
Arg128	12.097	>12.000
Tyr130	13.885	>12.000
Arg133	11.683	>12.000
His135	5.897	6.658
Lys136	9.607	10.193
Arg139	11.98	>12.000
Arg140	11.792	>12.000
Asp141	3.743	3.709
Asp142	2.908	2.316
Arg146	11.982	>12.000
Tyr152	11.455	>12.000
His154 *	5.189	<0.000
Tyr155	15.146	>12.000

His156 *	5.949	7.697
Asp157	3.834	<0.000
His158 *	6.174	8.231
Glu163	4.232	3.47
His164	6.131	6.065
Arg170	11.628	>12.000
Tyr174	15.183	>12.000
Arg180	10.246	>12.000
Arg181	11.308	>12.000
Lys182	10.059	10.184
Asp184	4.2	<0.000
Asp188	3.965	3.1
His191	5.299	6.34
Asp197	4.587	2.53
Arg203	11.999	>12.000
Lys204	10.497	10.774
His206	6.282	6.533
Asp210	4.514	3.464
Glu212	4.567	2.726
Asp217	4.077	3.038
Arg218	11.693	>12.000
Glu220	3.934	<0.000
His226	4.913	1.486
Glu228	4.408	4.002
Tyr229	9.814	10.88
Tyr230	10.223	10.587
His231	5.325	<0.000
His234 *	7.158	8.574
His236 *	6.259	4.37
His238	4.423	1.204
Arg239	12.182	>12.000
Asp242	4.767	2.171
Arg244	11.16	>12.000
Asp252	3.95	4.033
Asp253	2.964	2.133
Arg256	11.857	>12.000
Asp259	4.798	1.344
Lys261	9.982	>12.000
Asp267	4.591	3
Glu277	4.678	3.756
Tyr286	14.976	>12.000
His287 *	6.091	1.856
Cys288	11.2	>12.000
His289 *	7.247	6.976
His293	6.224	6.27
Asp295	4.417	3.428

Lys305	10.114	>12.000
Lys306	9.874	10.521
Asp308	3.297	2.545
Tyr314	10.263	11.367

Note 4: Calculated values are not real pKa values of residues, but can reflect the tendency of a residue to be protonated or deprotonated at specified pH. Ionizable key residues surrounding Ru-Cu BNC are marked by *.

Table S5. Statistics for crystallographic data collection and structure refinement.

Wavelength (Å)	0.9789
Resolution range (Å)	30.89 - 2.1 (2.175 - 2.1)
Space group	P 4332
Unit cell	177.441 177.441 177.441 90 90 90
Total reflections	
Unique reflections	55922
Completeness (%)	99.79 (100.00)
Redundancy	43.1（41.8）
Mean I/sigma(I)	21（1.2）
Wilson B-factor	38.98
R-merge	19.1 （327.1）
CC1/2	99.6（63.2）
Reflections used in the refinement	55916 (5490)
Reflections used for R-free	2817（281）
R-work	0.1728
R-free	0.1907
Number of non-hydrogen atoms	2435
macromolecules	2154
ligands	3
RMS (bonds)	0.014
RMS (angles)	2.23
Ramachandran favored (%)	97.47
Ramachandran allowed (%)	2.53
Ramachandran outliers (%)	0.00
Rotamer outliers (%)	1.35
Clashscore	2.86
Average B-factor	43.01
macromolecules	41.72
ligands	62.50
solvent	52.78
